# Plasma-Derived HIV-1 Virions Contain Considerable Levels of Defective Genomes

**DOI:** 10.1128/jvi.02011-21

**Published:** 2022-03-23

**Authors:** Katie Fisher, Xiao Qian Wang, Ashley Lee, Vincent Morcilla, Anneke de Vries, Eunok Lee, John-Sebastian Eden, Steven G. Deeks, Anthony D. Kelleher, Sarah Palmer

**Affiliations:** a Centre for Virus Research, The Westmead Institute of Medical Research, The University of Sydney, Sydney, New South Wales, Australia; b Sydney Medical School, Westmead Clinical School, Faculty of Medicine and Health, The University of Sydney, Sydney, New South Wales, Australia; c Division of HIV, Infectious Diseases and Global Medicine, Department of Medicine, Zuckerberg San Francisco General Hospital, University of California San Francisco, San Francisco, California, USA; d Kirby Institute, The University of New South Wales, Sydney, New South Wales, Australia; Emory University

**Keywords:** human immunodeficiency virus

## Abstract

Genetically-characterizing full-length HIV-1 RNA is critical for identifying genetically-intact genomes and for comparing these RNA genomes to proviral DNA. We have developed a method for sequencing plasma-derived RNA using long-range sequencing (PRLS assay; ∼8.3 kb from *gag* to the 3′ end or ∼5 kb from *integrase* to the 3′ end). We employed the *gag*-3′ PRLS assay to sequence HIV-1 RNA genomes from ART-naive participants during acute/early infection (*n* = 6) or chronic infection (*n* = 2). On average, only 65% of plasma-derived genomes were genetically-intact. Defects were found in all genomic regions but were concentrated in *env* and *pol*. We compared these genomes to near-full-length proviral sequences from paired peripheral blood mononuclear cell (PBMC) samples for the acute/early group and found that near-identical (>99.98% identical) sequences were identified only during acute infection. For three participants who initiated therapy during acute infection, we used the *int*-3′ PRLS assay to sequence plasma-derived genomes from an analytical treatment interruption and identified 100% identical genomes between pretherapy and rebound time points. The PRLS assay provides a new level of sensitivity for understanding the genetic composition of plasma-derived HIV-1 RNA from viremic individuals either pretherapy or after treatment interruption, which will be invaluable in assessing possible HIV-1 curative strategies.

**IMPORTANCE** We developed novel plasma-derived RNA using long-range sequencing assays (PRLS assay; 8.3 kb, *gag*-3′, and 5.0 kb, *int*-3′). Employing the *gag*-3′ PRLS assay, we found that 26% to 51% of plasma-derived genomes are genetically-defective, largely as a result of frameshift mutations and deletions. These genetic defects were concentrated in the *env* region compared to *gag* and *pol*, likely a reflection of viral immune escape in *env* during untreated HIV-1 infection. Employing the *int*-3′ PRLS assay, we found that analytical treatment interruption (ATI) plasma-derived sequences were identical and genetically-intact. Several sequences from the ATI plasma samples were identical to viral sequences from pretherapy plasma and PBMC samples, indicating that HIV-1 reservoirs established prior to therapy contribute to viral rebound during an ATI. Therefore, near-full-length sequencing of HIV-1 particles is required to gain an accurate picture of the genetic landscape of plasma HIV-1 virions in studies of HIV-1 replication and persistence.

## INTRODUCTION

The major barrier to an HIV-1 cure is the persistence of genetically-intact, and likely replication-competent, provirus in resting CD4^+^ T cells despite suppressive antiretroviral therapy (ART) (1–3). Upon the cessation of ART, plasma viremia rapidly returns to levels seen pre-ART (4–6), and determining the cellular source of these rebound viruses is imperative for the identification of targets for curative strategies. In addition, it is important to study the characteristics of pretherapy viremia, as the genetic composition of these plasma-derived virions can provide important information such as the replication rate and genetic diversity of HIV-1 *in vivo* (7–11), timing of the establishment of the latent reservoir that persists during ART (12–14), and the contribution of pretherapy virus to viral rebound after treatment interruption (15–17).

Some of the most widely used methods to study HIV-1 replication involve sequencing HIV-1 RNA from the plasma of HIV-1-infected individuals by using techniques such as single-genome sequencing (SGS) methods that amplify and sequence only a small region of the HIV-1 RNA genome (<1 to 2.5 kb) (8, 18, 19). These methods are high throughput and cost effective, can identify minority variants within a population, and give an accurate estimate of the genetic diversity of the viral population (7, 18, 20–22).

However, methods that sequence only a small region of the HIV-1 RNA genome miss crucial information outside of the sequenced region. It is well known that sequences that are genetically-intact or genetically-identical to another sequence in a small part of the HIV-1 RNA genome may not be intact or identical within the full-length genome due to defects or mutations outside of the sequenced region (23–25). While it has been observed in the past that some genetically-defective RNA genomes can be isolated from the plasma of HIV-1-infected individuals with or without ART (19, 26–29), the distribution of these defects is unknown due to a lack of studies sequencing the full-length plasma-derived HIV-1 RNA genome. Moreover, sequencing the entire HIV-1 RNA genome provides further information such as HLA-associated cytotoxic T lymphocyte (CTL) escape mutations and recombination events, both of which can be found in all regions of the HIV-1 genome, though with differing frequencies (30–32). Several groups have attempted to sequence the entire HIV-1 plasma-derived RNA genome using multiple overlapping fragments (30, 33–37). While these methods are able to capture near-full-length RNA genomes, some at a single-copy level (33, 37), it can be difficult to definitively determine which fragments form a single full-length HIV-1 RNA genome, as the region of overlap between the two amplified fragments is typically quite small.

In the present study, we developed a novel method to amplify and sequence plasma-derived HIV-1 RNA using long-range sequencing (PRLS assay). This assay allows for the sequencing and genetic characterization of near-full-length single amplicons (*gag*-3′, 8.3 kb) or amplicons spanning *integrase* to the 3′ end (*int*-3′, 5 kb). Employing the *gag*-3′ PRLS assay, we sequenced plasma-derived RNA genomes from untreated HIV-1-infected participants and identified that 26 to 51% of plasma-derived viral particles contain genetically-defective genomes, mostly resulting from frameshift mutations and large internal deletions. We also used the *int*-3′ PRLS assay to compare analytical treatment interruption (ATI) plasma-derived sequences from participants who initiated ART during acute infection to pre-ART plasma-derived and peripheral blood mononuclear cell (PBMC)-derived genomes and identified genetically-identical and intact genomes between these time points within the region spanning *int*-3′. Therefore, full-length sequencing of HIV-1 particles is required to gain an accurate picture of the genetic landscape of plasma HIV-1 virions in studies of HIV-1 replication and persistence.

## RESULTS

The single-genome sequencing (SGS) (18) and full-length individual proviral sequencing (FLIPS) (38) assays were modified to develop the plasma-derived HIV-1 RNA using the long-range sequencing (PRLS) assay. The PRLS assay involves extraction of viral RNA from plasma samples from viremic HIV-1-infected individuals, followed by cDNA synthesis of the full HIV-1 RNA template using an HIV-1-specific anchored oligo(dT) primer (39) and a reverse transcriptase that most efficiently synthesizes long cDNA fragments (AffinityScript multiple temperature reverse transcriptase; Agilent Technologies). The cDNA is then amplified using the modified FLIPS assay, which allows for sequencing of near-full-length individual plasma-derived HIV-1 RNA genomes, incorporating *gag* to the 3′ end (*gag*-3′; 8.3 kb). To overcome the reduced efficiency caused by incomplete reverse transcription in plasma samples with lower viral loads, we also developed a modified version of the PRLS assay that allows amplification and sequencing of the region incorporating *integrase* to the 3′ end (*int*-3′; 5 kb).

### Determination of PRLS assay error rate.

To assess the extent of nucleotide misincorporation (polymerase errors) that may occur during the PRLS assay, we activated J-Lat 10.3 cells (40) with phorbol 12-myristate 13-acetate (PMA) and extracted the supernatant before amplifying the cDNA with the *gag*-3′ primers. We compared 94 individual *gag*-3′ sequences (828,705 total nucleotides) to the published sequence of the proviral genome found in J-Lat cells (41) and determined the overall error rate of the assay per nucleotide to be 0.0076% (95% confidence interval [CI], 0.0054%, 0.0098%), or approximately 0.63 nucleotides/*gag*-3′ genome.

To determine the rate of assay-related intertemplate recombination introduced during reverse transcription, plasma samples from two different participants (participants P6 and P7, with distinct viral sequences) containing approximately 12,000 HIV-1 RNA copies were mixed prior to RNA extraction and *gag*-3′ sequencing. We observed no evidence of intertemplate recombination from 87 sequences (708,560 nucleotides) amplified from the mixed extraction. We therefore estimated the rate of *in vitro* recombination within each RNA template to be <1.4 × 10^−6^ per nucleotide.

### PRLS assay lower limit.

**FIG 1:**
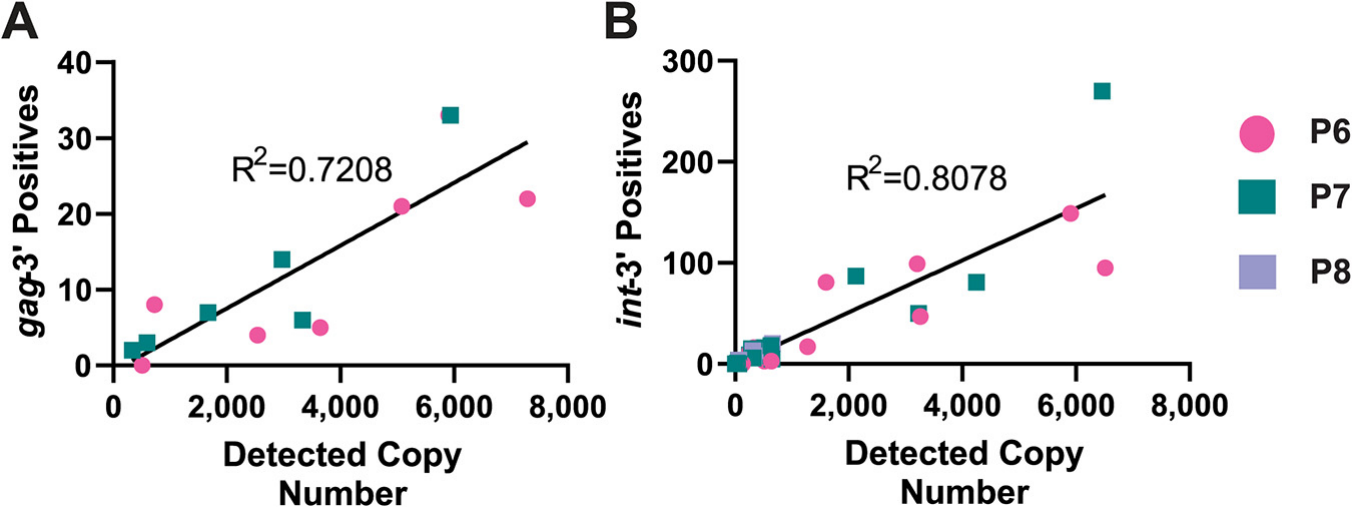
Lower limits of PRLS assay. Plasma samples were diluted to a range of different copy numbers, followed by sequencing of amplified positives. The number of positives obtained per dilution was plotted against the copy number in the extraction detected by ddPCR. A simple linear regression was calculated to determine the reproducibility of the assay for each diluted plasma sample. (A) For the *gag*-3′ assay, plasma samples from participants P6 and P7 were diluted to copy numbers ranging from >10,000 copies to <1,000 copies. (B) For the *int*-3‘assay, plasma samples from participants P6, P7, and P8 were diluted to copy numbers ranging from 10,000 copies to <100 copies.

To determine the lower limits and reproducibility of the PRLS assay for both the *gag*-3′ and *int*-3′ amplicons, plasma samples from 2 or 3 untreated HIV-1-infected participants were diluted from 10,000 copies to 1,000 copies for the *gag*-3′ assay and from 10,000 copies to <100 copies for the *int*-3′ assay. Each dilution was run in duplicate per participant. The actual copy number extracted was measured by droplet digital PCR (ddPCR), and this was plotted against the number of amplicons obtained from the extraction (Fig. 1). For the *gag*-3′ assay, the lower limit of the assay was calculated to be 350 copies, with amplicons consistently obtained for samples with viral loads of >500 copies (Fig. 1A). For the *int*-3′ assay, the lower limit of the assay was determined to be 40 copies, with amplicons consistently obtained for samples with viral loads of >100 copies (Fig. 1B).

**FIG 2:**
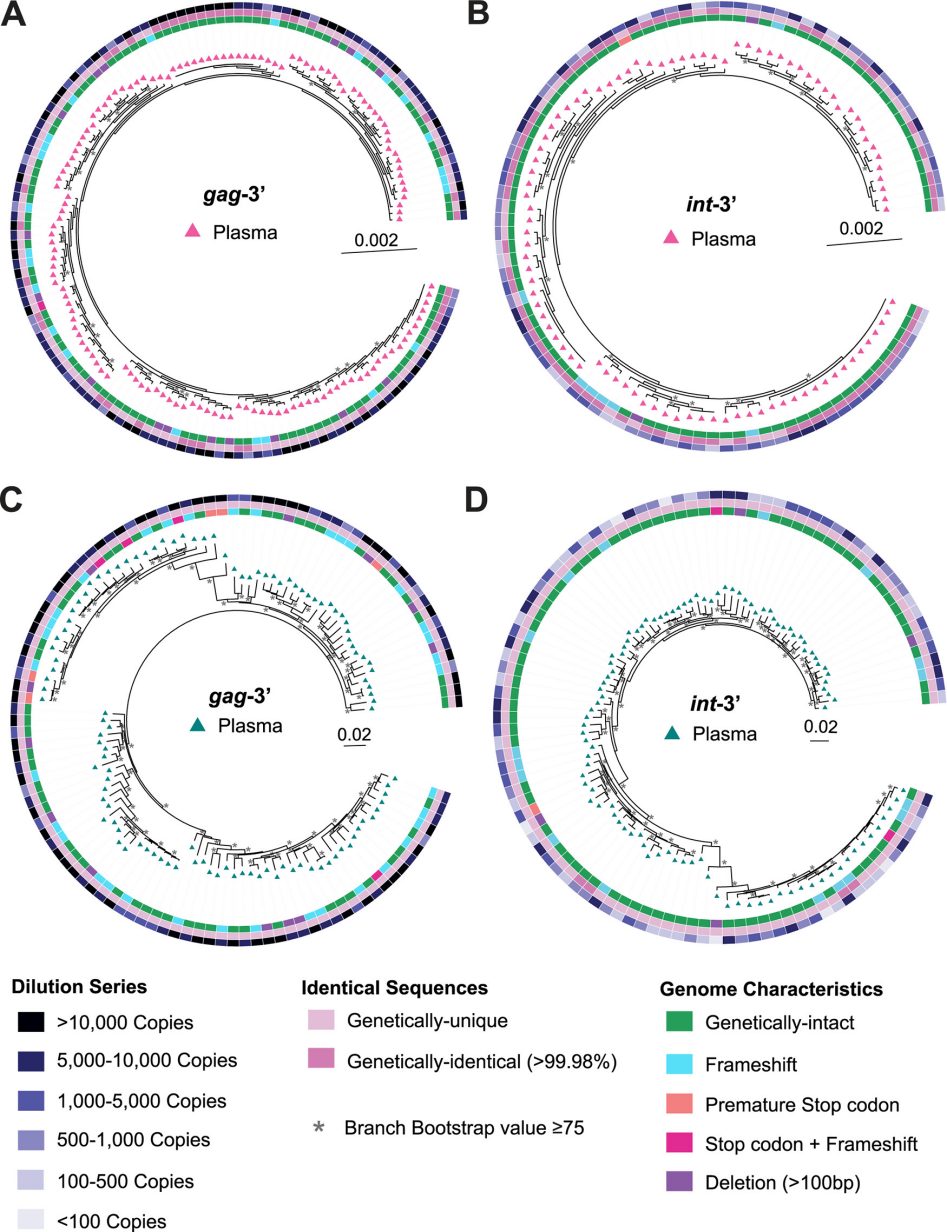
Phylogeny of sequenced plasma-derived genomes from diluted plasma. Pre-ART plasma samples from participants P6 and P7 were diluted to copy numbers ranging from >10,000 copies to <1,000 copies for the *gag*-3′ assay and from 10,000 copies to <100 copies for the *int*-3′ assay, followed by sequencing of the amplified positives. Phylogenetic trees were prepared from the sequenced *gag*-3′ (A and C) and *int*-3′ (B and D) genomes for participants P6 (A and B) and P7 (C and D). Outer rings of the trees show the estimated copy number of the sample from which the sequence was amplified, middle rings show whether the sequence is >99.98% identical to another sequence in the tree, and inner rings show whether the sequences are classified as genetically-intact or defective within the sequenced region with each type of defect shown. All sequences on the trees are plasma-derived, as indicated by triangles. Branches with bootstrap values of ≥75 are indicated by an asterisk beside the node.

To ensure that diluting the plasma samples did not cause selection for specific variants within each plasma sample, we constructed a phylogenetic tree containing genomic sequences obtained from each dilution, as well as from extractions of >10,000 copies for the *gag*-3′ amplicon, for two of the participant samples used to quantify the lower limits of the assay (P6 and P7) (Fig. 2). For both participants and both regions (*gag*-3′ or *int*-3′), there was no evidence of selection for specific genomic variants, as sequences from each dilution intermingled within the trees (outer rings of the phylogenetic trees). We also calculated the percent genetic diversity of the genetically-intact sequences by using average pairwise distance (APD). The percentages of genetic diversity of the sequences from each dilution for each participant were similar, further indicating that there was no sequencing-related bias within the dilutions (Table 1).

**TABLE 1:** Genetic diversity of *gag*-3′ and *int*-3′ intact sequences across plasma dilutions for P6 and P7

Participant	Diluted copy no.	*gag*-3′		*int-3′*	
		No. of intact sequences	Genetic diversity (APD) (%)	No. of intact sequences	Genetic diversity (APD) (%)
P6	All intact sequences	98	0.05	84	0.06
	10,000	47	0.06	18	0.05
	5,000	6	0.07	20	0.06
	1,000	11	0.04	32	0.07
	500	NA^*a*^	NA	14	0.05
	100	NA	NA	0	NA
P7	All intact sequences	54	2.21	78	2.63
	10,000	13	2.16	13	2.85
	5,000	10	2.27	17	2.64
	1,000	3	NA	22	2.62
	500	NA	NA	25	2.61
	100	NA	NA	3	NA

a NA, not applicable.

### Defective HIV-1 genomes are present in plasma-derived virions.

**FIG 3:**
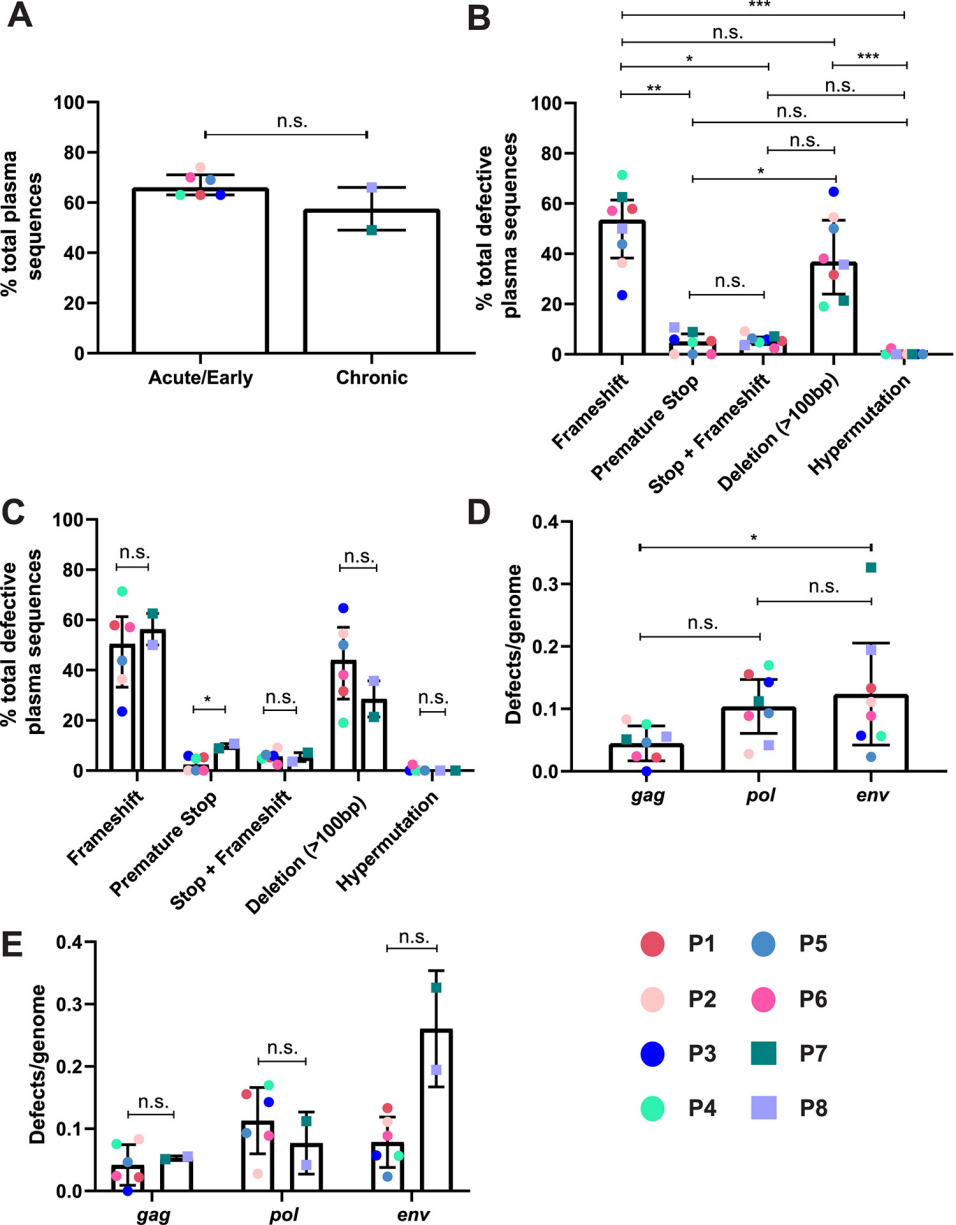
Types of defects present in the plasma of ART-naive HIV-1-infected individuals. *gag*-3′ RNA genomes were amplified using PRLS and sequenced from the plasma of 8 ART-naive HIV-1-infected participants. The acute/early participants (*n* = 6) are depicted by circles, and the chronic participants are depicted by squares (*n* = 2). (A) Proportion of total plasma-derived *gag*-3′ sequences that were genetically-intact in the acute/early group versus the chronic group. (B and C) Proportions of total genetically-defective plasma-derived *gag*-3′ genomes with different types of genetic defects: acute/early and chronic groups combined (B) or separated (C). Data are represented as the median ± IQR. (D and E) For full-length plasma-derived *gag*-3′ genomes that lack deletions >100 bp in size, the number of defects (premature stop codons and frameshift mutations combined) found in *gag*, *pol*, or *env* was divided by the number of full-length genomes amplified per participant. This was compared between these three genomic regions for the acute/early and chronic participant groups combined (D) and separately (E). Data are represented as the mean ± SD. *, *P* < 0.05; **, *P* < 0.01; ***, *P* < 0.001; n.s., *P* > 0.05.

It has generally been assumed that the majority of virions within the plasma contain genetically-intact HIV-1 RNA genomes. To investigate the number of genetically-defective and intact HIV-1 RNA genomes within the plasma of untreated HIV-1-infected participants, we used the *gag*-3′ PRLS assay to sequence HIV-1 RNA from the plasma of eight ART-naive HIV-1-infected participants. The plasma samples were collected during acute/early infection and chronic infection for participants P1 to P6 and participants P7 and P8, respectively. Participant characteristics and numbers of sequences amplified are shown in Table 2. We found an overall median of 65% (interquartile range [IQR], 63 to 69%) of plasma RNA genomes were genetically-intact in the *gag*-3′ region. When the proportion of genetically-intact RNA sequences from the plasma of the acute/early participants was compared with that of the chronically-infected participants, we found that there was no significant difference between these participant groups. The acute/early group had a median of 66% (IQR, 63 to 70%) of genetically-intact genomes, compared to a median of 58% (IQR, 53 to 62%) for the chronic group (Fig. 3A) (*P* = 0.31).

**TABLE 2:** Number of sequences amplified per sample

Sample type and participant	Sample time point	Stage of infection	Estimated time of infection at time of blood draw (pre-ART samples)	Viral load of plasma (copies/mL) or no. of PBMCs used for sequencing	Assay	No. of genetically-intact sequences	No. of defective sequences with:						Total no. of sequences
							Frameshift mutation	Stop codon	Stop codon + frameshift	Deletion	Hypermutation	*cis*-acting defect	
Pre-ART plasma sequences (all sequence lengths)													
P1	Pre-ART	Early	4.5 mo	750,000	*gag*-3′ PRLS	32	11	1	1	6	0	NA^*a*^	51
P2	Pre-ART	Early	4.25 mo	750,000	*gag*-3′ PRLS	31	4	0	1	6	0	NA	42
P3	Pre-ART	Acute	1 mo	750,000	*gag*-3′ PRLS	29	4	1	1	11	0	NA	46
P4	Pre-ART	Acute	1.25 mo	125,000	*gag*-3′ PRLS	36	15	1	1	4	0	NA	57
P5	Pre-ART	Acute	1.5 mo	65,000	*gag*-3′ PRLS	35	7	0	1	8	0	NA	51
P6	Pre-ART	Acute	1 mo	3,794,000	*gag*-3′ PRLS	98	24	0	1	16	1	NA	140
P7 TP1	Pre-ART	Chronic	>1 yr	300,000	*gag*-3′ PRLS	54	35	5	4	12	0	NA	110
P7 TP2	Pre-ART	Chronic	>1 yr	210,000	*gag*-3′ PRLS	46	25	3	2	9	1	NA	86
P8	Pre-ART	Chronic	>1 yr	645,000	*gag*-3′ PRLS	54	14	3	1	10	0	NA	82

Pre-ART PBMC proviral sequences (>8,800 bp only)													
P1	Pre-ART	Early	4.5 mo	306,173	FLIPS	37	4	3	0	NA	4	0	48
P2	Pre-ART	Early	4.25 mo	488,148	FLIPS	42	2	1	0	NA	5	0	50
P3	Pre-ART	Acute	1 mo	553,086	FLIPS	28	2	2	0	NA	5	0	37
P4	Pre-ART	Acute	1.25 mo	1,441,975	FLIPS	21	1	1	0	NA	6	1	30
P5	Pre-ART	Acute	1.5 mo	1,975,309	FLIPS	22	2	1	0	NA	1	0	26
P6	Pre-ART	Acute	1 mo	464,198	FLIPS	66	3	0	1	NA	1	0	74

ATI plasma sequences (all sequence lengths)													
P3	ATI (B1 Wk1)	Acute treatment	NA	920	*int*-3′ PRLS	37	3	0	0	1	0	NA	41
P4	ATI (B2 Wk4)	Acute treatment	NA	1,081	*int*-3′ PRLS	23	4	0	0	2	0	NA	29
P5	ATI (B2 Wk2)	Acute treatment	NA	1,200	*int*-3′ PRLS	36	3	0	0	0	0	NA	39

a NA, not applicable.

Overall, we found that frameshift mutations were the most common type of genetic defect found, making up an overall median of 54% (IQR, 42 to 59%) of the total number of defective plasma-derived genomes amplified, followed by deletions of >100 bp (median, 37%; IQR, 29 to 51%) (Fig. 3B). Other defects such as single nucleotide polymorphisms (SNPs) causing premature stop codons and hypermutations had a lower frequency (median, <5.5%; range, 0 to 11%). When comparing the data for the acute/early versus chronic participants, we found weak evidence that the proportion of sequences containing a premature stop codon was higher in the chronically-infected participants than in the acute/early participants (Fig. 3C) (*P* = 0.04). We found no statistically significant difference in the proportion of sequences with any other type of defect between these two participant groups (Fig. 3C) (*P* > 0.32).

We also investigated whether these genetic defects are concentrated in any HIV-1 open reading frame (ORF). When pooling only the full-length *gag*-3′ sequences (length, >8,250 bp) across all participants, we found that the total number of defects (frameshift and stop codons) was highly concentrated in *env* (49.5% of defects), followed by *pol* (29.2% of defects) (Table 3). When the number of defects in each gene was taken as a proportion of the gene length to account for the differing lengths of the HIV-1 ORFs, the most defects were still found in *env*, followed by *pol* (Table 4).

**TABLE 3:** Number of defects within each HIV-1 gene of sequenced *gag*-3′ genomes

Gene	Participant group	Total no. of frameshifts	Total no. of stop codons	Total no. of defects from full-length sequences
*gag*	All	24	0	24
	Acute/early	13	0	13
	Chronic	11	0	11
*pol*	All	48	11	59
	Acute/early	32	5	37
	Chronic	16	6	22
*vif*	All	9	1	10
	Acute/early	9	0	9
	Chronic	0	1	1
*vpr*	All	3	1	4
	Acute/early	1	0	1
	Chronic	2	1	3
*tat*	All	3	0	3
	Acute/early	2	0	2
	Chronic	1	0	1
*rev*	All	0	0	0
	Acute/early	0	0	0
	Chronic	0	0	0
*vpu*	All	1	1	2
	Acute/early	1	0	1
	Chronic	0	1	1
*env*	All	84	16	100
	Acute/early	21	6	27
	Chronic	63	10	73

**TABLE 4:** Defects found across the sequenced *gag*-3′ HIV-1 genome as a proportion of the length of the gene

Gene	Participant group	No. of frameshifts/length of gene^*a*^	No. of stop codons/length of gene^*a*^	Total no. defects/length of gene^*a*^
*gag*	All	0.016	0	0.016
	Acute/early	0.009	0	0.009
	Chronic	0.007	0	0.007
*pol*	All	0.016	0.003	0.020
	Acute/early	0.011	0.002	0.012
	Chronic	0.005	0.002	0.007
*vif*	All	0.016	0.002	0.017
	Acute/early	0.016	0	0.016
	Chronic	0	0.002	0.002
*vpr*	All	0.01	0.003	0.014
	Acute/early	0.003	0	0.003
	Chronic	0.007	0.003	0.01
*tat*	All	0.01	0	0.01
	Acute/early	0.007	0	0.007
	Chronic	0.003	0	0.003
*rev*	All	0	0	0
	Acute/early	0	0	0
	Chronic	0	0	0
*vpu*	All	0.004	0.004	0.008
	Acute/early	0.004	0	0.004
	Chronic	0	0.004	0.004
*env*	All	0.033	0.006	0.039
	Acute/early	0.008	0.002	0.011
	Chronic	0.025	0.004	0.028

a Gene length is measured in bp.

To further investigate whether *gag*, *pol*, or *env* contained more defects in full-length (>8,250 bp) sequences, we adjusted the number of frameshifts or stop codons to the number of genomes analyzed in that gene region per participant (Fig. 3D and E). We found that *gag* had a lower mean value for defects/genome than *env* (Fig. 3D) (*P* = 0.032). We also observed that the mean defects/genome were lower in *gag* than in *pol* (0.045 and 0.104 defects/genome, respectively), though this did not reach statistical significance (Fig. 3D) (*P* = 0.328). When comparing acute/early infection and chronic infection, we observed a trend toward chronically-infected participants having a higher mean number of defects/genome in the *env* region than acute/early participants (Fig. 3E) (*P* = 0.147). However, there was no difference in the number of defects/genome in *gag* and *pol* between the two participant groups (*P* > 0.9 for both). We note that the *gag*-3′ PRLS assay does not amplify the first nine codons of *gag*, meaning that we could miss changes in the *gag* start codon that render a genome defective.

Interestingly, out of 82 total sequences with deletions (>100 bp) identified in the plasma from the eight ART-naive participants (Table 2), we found that seven (8.5%), sequenced from five different participants, had deletion breakpoints that corresponded to the splice donor site D4 (HXB2 positions 6043 to 6053) and acceptor site A7 (HXB2 positions 8359 to 8381) (42). Splicing at these sites joins *tat* and *rev* exons 1 and 2. In addition, five sequences were identified with 3′ deletion breakpoints corresponding to known splice acceptor sites (42), even though the 5′ breakpoints of some did not correspond to a known splice donor site.

### Genetic diversity of plasma genomes during untreated HIV-1 infection.

**FIG 4:**
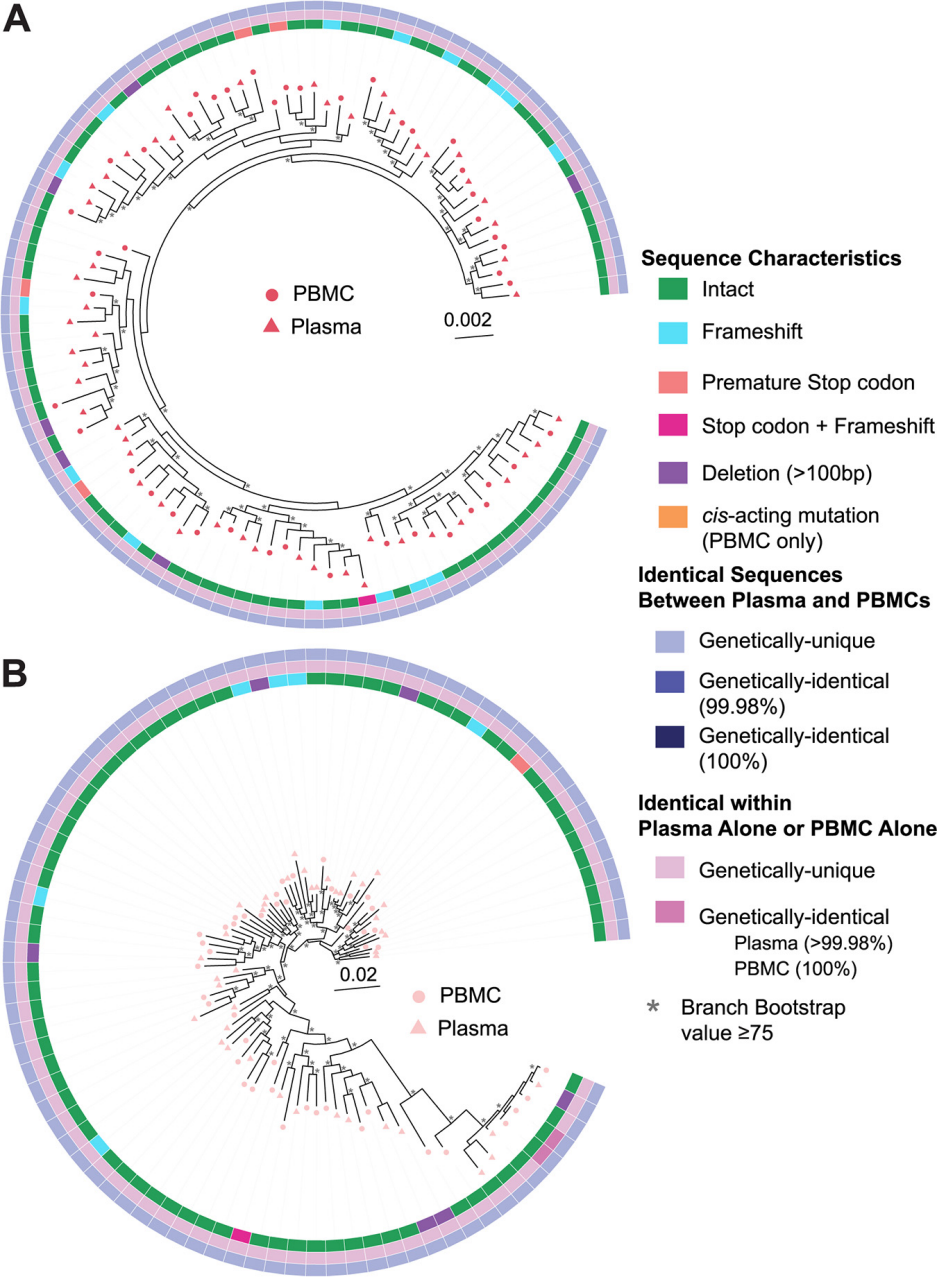
Phylogenetic trees of pre-ART plasma- and PBMC-derived sequences for the early infection group. All pre-ART plasma- and PBMC-derived sequences, excluding hypermutated sequences, were trimmed and aligned in the *gag*-3′ region, and phylogenetic trees were prepared for participants P1 (A) and P2 (B). PBMC-derived sequences are represented by circles, while plasma-derived sequences are represented by triangles. Inner rings show whether a sequence is genetically-intact or defective for each type of defect shown. PBMC-derived sequences are classified as intact or defective based on the FLIPS 9 kb sequence, while plasma-derived sequences are classified as intact or defective in the *gag*-3′ region (8.3 kb). Middle rings indicate whether a sequence is identical to another sequence only within the plasma or PBMC compartments. PBMC-derived sequences are classified as identical to another PBMC sequence if they are 100% identical in the *gag*-3′ region, while plasma-derived sequences are classified as identical to another plasma sequence if they are >99.98% identical to another sequence. Outer rings indicate whether a plasma sequence is 99.98% identical or 100% identical to a PBMC sequence. Branches with bootstrap values of ≥75 are indicated by an asterisk beside the node.

Studies sequencing shorter HIV-1 RNA fragments have revealed that during untreated acute/early infection, HIV-1 RNA is genetically homogenous (8, 22, 43–46). To determine if the genetic diversity of near-full-length genetically-intact sequences is different during acute/early and chronic infection, we calculated the genetic diversity of *gag*-3′ plasma RNA sequences for each participant using average pairwise distance (APD). Similar to these earlier findings (8, 22, 43–46), the genetically-intact HIV-1 RNA genomes within the majority of the acute/early participants were less genetically diverse than those of the chronic participants (acute/early group = 0.05 to 1.72%, chronic group = 1.11 to 2.25%) (Table 5). However, the APD of the genetically-intact HIV-1 RNA genomes derived from participant P2, whose plasma sample was collected during early infection, was 1.72% (Table 5). This high genetic diversity and the topology of the phylogenetic tree observed for this participant (Fig. 4B) are consistent with previous reports of early infection with multiple variants (7, 8, 22). Participant P7, whose sample was collected during chronic infection, also had an extremely diverse population of genetically-intact genomes isolated from their plasma (APD = 2.25%) (Table 5). Although genetic diversity is expected to increase during untreated chronic infection (8, 22, 45, 46), the phylogenetic tree of this participant indicates infection with multiple variants, as shown by the distance between branches of the viral sequences on the tree (Fig. 2C).

**TABLE 5:** Genetic diversity and proportion of clonal sequences in plasma during untreated HIV-1 infection

Participant	Stage of infection	Amplification assay	Genetic diversity of intact sequences (%)	99.98% identity	
				% of clonal sequences	No. of clones
P1	Early	*gag*-3′ PRLS	0.31	0	0
P2	Early	*gag*-3′ PRLS	1.72	0	0
P3	Acute	*gag*-3′ PRLS	0.06	13.04	2
P4	Acute	*gag*-3′ PRLS	0.10	12.3	1
P5	Acute	*gag*-3′ PRLS	0.08	9.8	1
P6	Acute	*gag*-3′ PRLS	0.05	32.1	2
P7	Chronic	*gag*-3′ PRLS	2.25	0	0
P8	Chronic	*gag*-3′ PRLS	1.11	31.7	4

**FIG 5:**
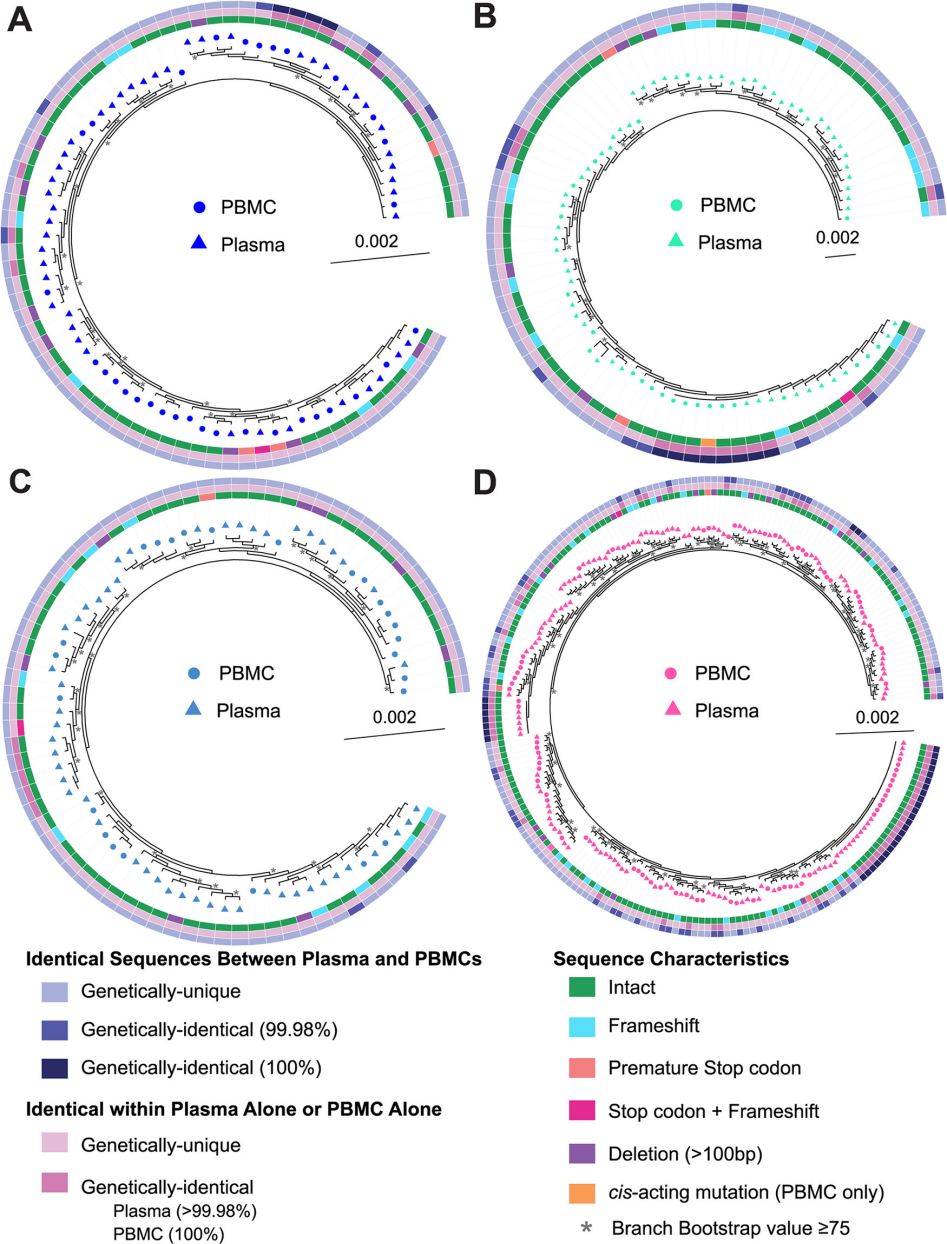
Phylogenetic trees of pre-ART plasma- and PBMC-derived sequences for the acute infection group. All pre-ART plasma- and PBMC-derived sequences, excluding hypermutated sequences, were trimmed and aligned in the *gag*-3′ region, and phylogenetic trees were prepared for participants P3 (A), P4 (B), P5 (C), and P6 (D). PBMC-derived sequences are represented by circles, while plasma-derived sequences are represented by triangles. Inner rings show whether a sequence is genetically-intact or defective for each type of defect shown. PBMC-derived sequences are classified as intact or defective based on the FLIPS 9 kb sequence, while plasma-derived sequences are classified as intact or defective in the *gag*-3′ region (8.3 kb). Middle rings indicate whether a sequence is identical to another sequence only within the plasma or PBMC compartments. PBMC-derived sequences are classified as identical to another PBMC sequence if they are 100% identical in the *gag*-3′ region, while plasma-derived sequences are classified as identical to another plasma sequence if they are >99.98% identical to another sequence. Outer rings indicate whether a plasma sequence is 99.98% identical or 100% identical to a PBMC sequence. Branches with bootstrap values of ≥75 are indicated by an asterisk beside the node.

**FIG 6:**
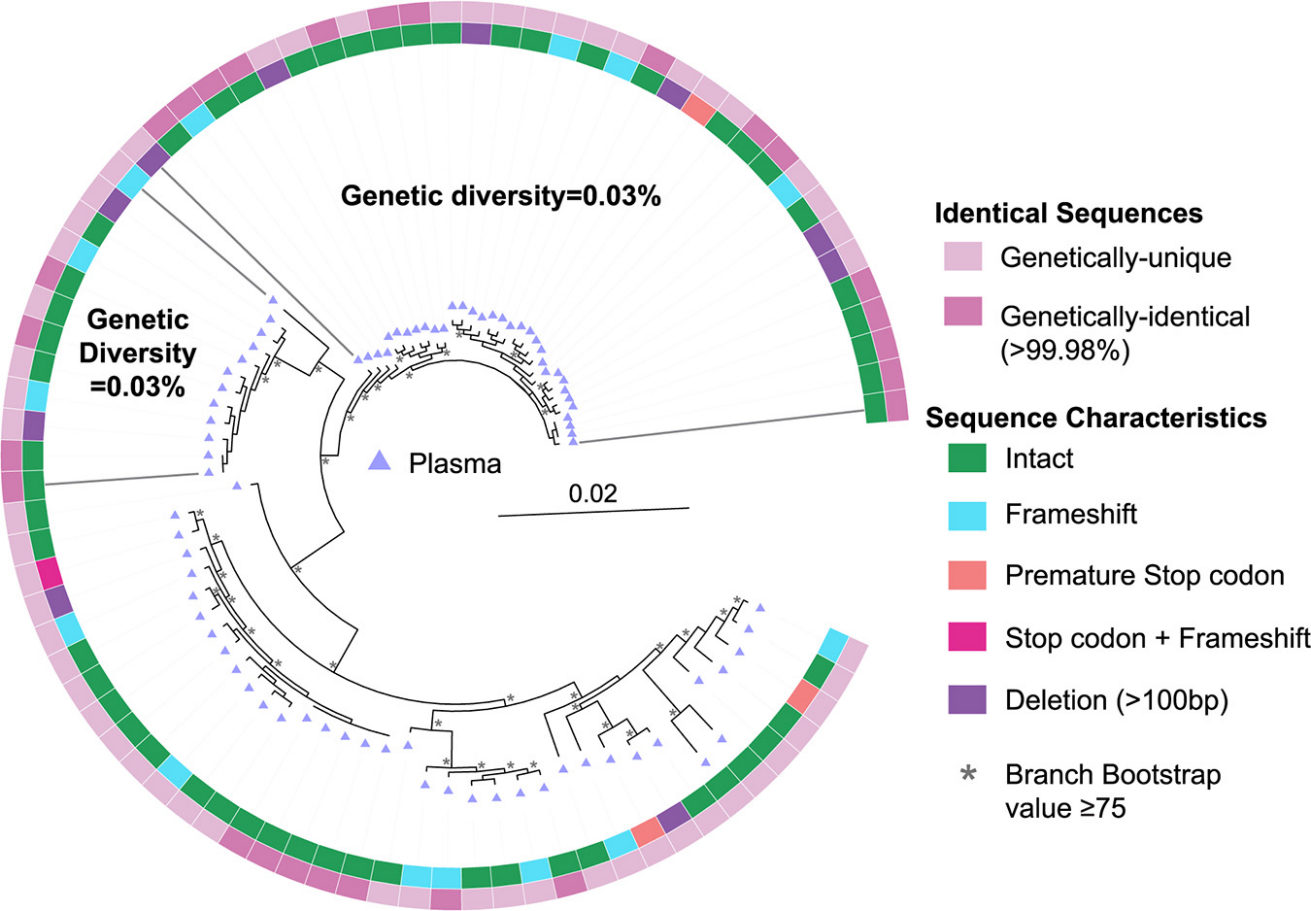
Phylogenetic trees of pre-ART plasma-derived sequences for participant P8. All pre-ART plasma-derived *gag*-3′ sequences were aligned, and phylogenetic trees were prepared for participant P8. Inner rings show whether a sequence is genetically-intact or defective for each type of defect shown. Outer rings show whether a sequence is >99.98% identical to another sequence on the tree. All sequences on the tree are plasma-derived, as indicated by the triangles. The genetic diversity of genetically-intact sequences within the most homogenous parts of the tree are shown between the dark gray lines. Branches with bootstrap values of ≥75 are indicated by an asterisk beside the node.

The presence of identical, or monotypic, plasma-derived genomes suggests that these genomes are part of a clone and were released as part of the same replication event, perhaps from the same infected cell or multiple infected cells with identical proviruses (46). We therefore investigated the proportion of genomes that are part of a clone within the plasma during untreated HIV-1 infection by using the *gag*-3′ assay. For this analysis, identical sequences that are part of a clone are >99.98% identical, which allows a difference of 1 nucleotide (nt) between full-length *gag*-3′ genomes. This was to allow for differences introduced during a single round of viral replication (47) or assay-related error. We identified plasma clones in the *gag*-3′ region in 5 of 8 participants (Table 5; Fig. 2C and Fig. 4 to 6). The proportion of plasma sequences that were part of a clone was low, ranging from 9.8% to 32%. As expected, the participants in earlier stages of untreated infection (Table 2), or those with lower genetic diversity, were more likely to have plasma clones that were part of only 1 or 2 clonal clusters. However, for P8, whose sample was collected during chronic infection, a high proportion of clonal plasma sequences within the *gag*-3′ region was identified (Table 5). These clonal sequences were part of 4 separate clonal clusters, 2 of which were found in regions of the phylogenetic tree with low genetic diversities of 0.03% (Table 5; Fig. 6). These results indicate that at least 4 different cells or groups of expanded cells contributed to viremia in this participant, similar to what has been identified previously during ART or ART interruption (48, 49). Importantly, across all participants, only 2 of 250 (0.8%) defective *gag*-3′ plasma genomes, both derived from participant P8, were 99.98% identical to a genetically-intact plasma genome, indicating that most errors are unique. Similarly, no sequences with deletions, including those with breakpoints coinciding with the D4 splice donor and A7 splice acceptor, were 99.98% identical to another short sequence. This indicates that these partially-spliced genomes could result from different replication events.

To investigate whether short HIV-1 RNA regions are predictive of the clonality of the full-length HIV-1 genome, we trimmed sequences amplified using the *gag*-3′ PRLS assay to the primer binding sites for *env* (V1-V3; ∼800 bp) and *p6-RT* (∼1.3 kb), which have been commonly used to sequence plasma HIV-1 RNA genomes (8, 17, 50, 51). We identified the proportion of clonal genomes within these trimmed alignments and compared that to the proportion of clonal genomes in the full *gag*-3′ alignments. For simplicity, we identified clonal genomes as those that were 100% identical to another genome for this analysis. The proportion of clonal sequences was much higher for the *env* and *p6-RT* primer sets than for *gag*-3′ for the two participants analyzed (Table 6). For participant P6, the proportion of clonal genomes increased from 13.6% to 82.4% when the *gag*-3′ sequences were trimmed to the *env* primers, and to 65.7% when the sequences were trimmed to the *p6-RT* primers. For participant P7, there were no clonal *gag*-3′ sequences, but when these sequences were trimmed to the *env* primer binding sites, 21% of sequences were found to be clonal, and 15% of sequences were clonal when trimmed to the *p6-RT* primer binding sites. This indicates that the *gag*-3′ primer set has a better capacity to correctly identify clonal genomes.

**TABLE 6:** Clonal sequences identified by *gag*-3′ PRLS, *env*, and *p6-RT*

Participant	Stage of infection	Amplified *gag*-3′ PRLS sequences		*env*		*p6-RT*	
		No. of sequences amplified	% Clonal	No. of sequences	% Clonal	No. of sequences	% Clonal
P6	Acute	140	13.6	131	82.4	134	65.7
P7	Chronic	110	0	106	20.8	103	14.6

### Longitudinal sample.

**FIG 7:**
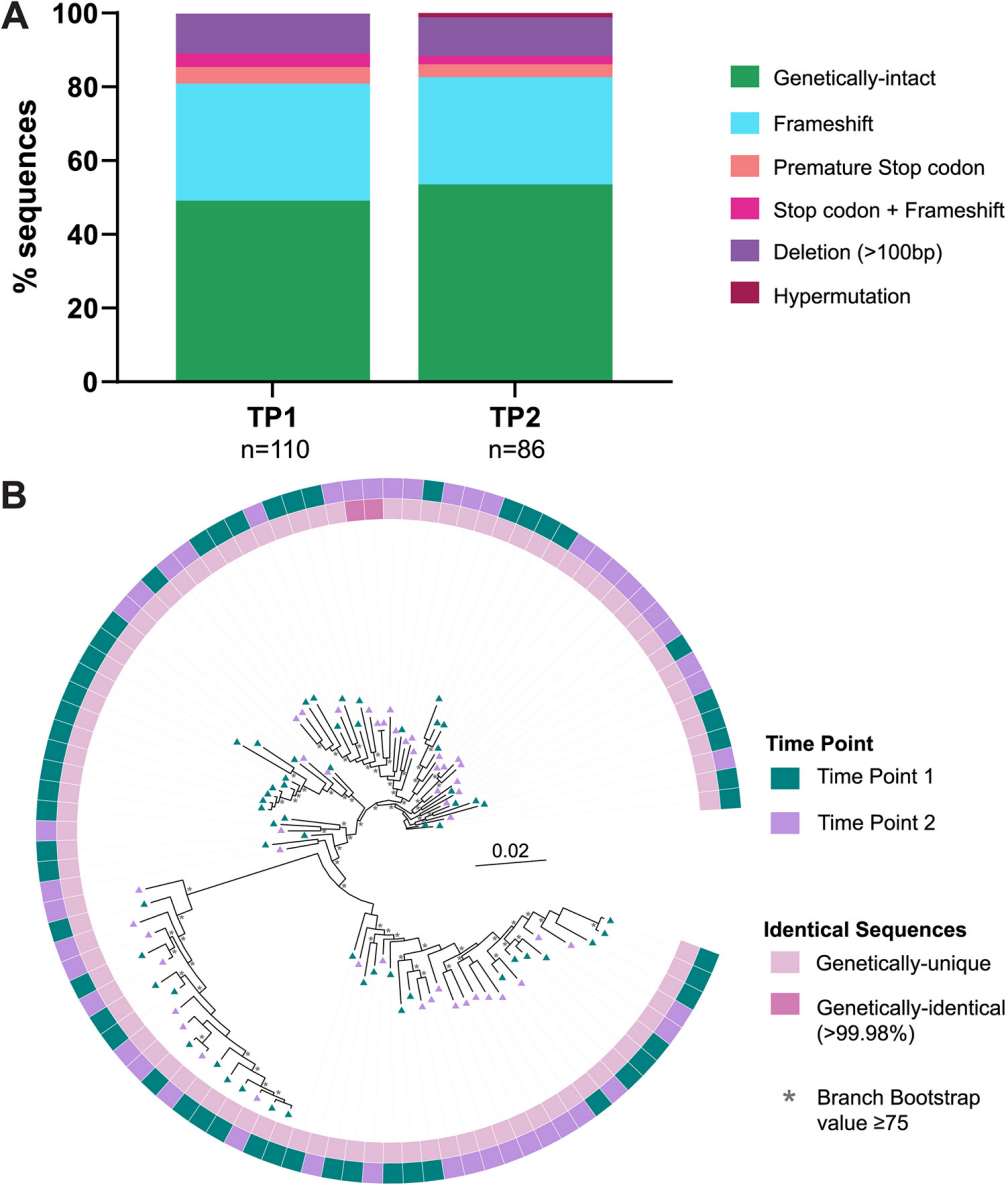
Genetic composition of plasma-derived *gag*-3′ PRLS sequences over 27 days of chronic untreated HIV-1 infection. For participant P7, plasma samples were available for time point 1 (TP1) and TP2, taken ∼27 days later. (A) The proportions of plasma-derived *gag*-3′ genomes that were genetically-intact or defective were compared between the two time points. *n* = number of *gag*-3′ genomes amplified. (B) A phylogenetic tree of all genetically-intact sequences amplified from P7 TP1 and TP2, with the outer ring and sequence symbols depicting the time point from which the sample came, and the inner ring showing which sequences were >99.98% identical to another sequence. All sequences on the tree are plasma-derived, as indicated by the triangles. Branches with bootstrap values of ≥75 are indicated by an asterisk beside the node.

To investigate if the genetic landscape of HIV-1 RNA virions changes over time during untreated chronic infection, we used the *gag*-3′ PRLS assay to sequence HIV-1 RNA genomes from two different plasma samples for P7, collected 27 days apart (time point 1 [TP1] and TP2). The percentages of sequences identified as genetically-intact were similar between the two time points (49% versus 54%), and the percentages of the different defects also remained similar between the two time points (Fig. 7A). The genetic diversity of intact sequences within the *gag*-3′ region did not change (2.3% for TP1, 2.2% for TP2). Similarly, the genetic diversity of intact sequences did not change substantially within specific gene regions (*gag*, TP1 = 1.73% and TP2 = 1.42%; *pol*, TP1 = 1.75% and TP2 = 1.59%; *env*, TP1 = 3.46% and TP2 = 3.4%). The sequences from the two time points were similar to one another and intermingled within the phylogenetic tree (Fig. 7B). This was confirmed using a test for panmixia, which indicates the probability that two groups of sequences come from two genetically distinct populations (52, 53). We used a threshold of *P* < 10^−3^ to indicate a significant genetic difference between the two groups (52, 54). We found no evidence of compartmentalization between the sequences from these two time points (*P* > 0.2). However, no identical *gag*-3′ sequences were identified across TP1 and TP2 (Fig. 7B, inner ring), indicating that despite the genetic similarity of the plasma virions, the continued replication during untreated infection has changed the genomic landscape between these two time points. Interestingly, P7 appeared to be infected with multiple variants at TP1 (Table 5; Fig. 2C), and these variants were also represented in the sequences from TP2 (Fig. 7B).

### Plasma sequences are similar to proviral PBMC sequences during untreated HIV-1 infection.

Comparing plasma-derived HIV-1 RNA genomes of viremic participants to proviral sequences obtained from cellular compartments may indicate the cellular source of this viremia. For participants P1 to P6, whose samples were collected during untreated acute/early infection, PBMC samples were available from the same blood draw as the plasma samples. We therefore used FLIPS (38) to genetically-characterize the proviruses present in the peripheral blood of these participants. We then compared these proviruses to the plasma-derived genomes from the same time point. For simplicity, we compared only full-length sequences for this analysis (sequences lacking deletions of >100 bp). The number of sequences amplified and the number of PBMCs analyzed per participant are shown in Table 2.

We next investigated whether identical sequences could be found between the plasma and PBMC compartments, setting the threshold for genetic identity at 99.98%, as described above. Genetically-identical sequences between the plasma and PBMCs in the *gag*-3′ region were identified in the four participants whose blood draw was taken during untreated acute infection (P3 to P6), with no identical sequences identified for the early infection participants (P1 and P2) (Table 7). For the four acute infection participants, between 1 and 42 plasma-derived RNA sequences were >99.98% identical to at least one proviral DNA sequence and represented 2 to 34% of the plasma-derived RNA sequences. For all participants, the identical sequences between the plasma and PBMC compartments were found in a single cluster of >99.98% sequences. Interestingly, 3 out of 4 of these near-identical sequence clusters contained multiple plasma and proviral sequences that were 100% identical in the *gag*-3′ region (Table 7), with most other sequences in the cluster differing from these by 1 nucleotide. Importantly, we found that none of the defective plasma-derived genomes were >99.98% identical to a proviral sequence.

**TABLE 7:** Genetic diversity and identical sequences across plasma and PBMCs during untreated HIV-1 infection

Participant	Stage of infection	Estimated time of infection at time of blood draw (mo)	% Genetic diversity in intact sequences in plasma (8.3 kb *gag*-3′ PRLS region)	% Genetic diversity in intact sequences in PBMCs (9.2 kb FLIPS region)	No. of clusters with >99.98% identical plasma and PBMC sequences	No. of *gag*-3′ plasma sequences		No. of PBMC sequences in cluster		Total no. of full-length *gag*-3′ plasma sequences available	Total no. of full-length PBMC sequences available
						Total no. >99.98% identical to a PBMC sequence	No. 100% identical to a PBMC sequence	Total no. >99.98% identical to a plasma sequence	No. 100% identical to a plasma sequence		
P1	Early	4.5	0.31	0.36	0	0	0	0	0	45	48
P2	Early	4.25	1.72	1.74	0	0	0	0	0	36	50
P3	Acute	1	0.06	0.06	1	4	2	5	2	35	37
P4	Acute	1.25	0.10	0.06	1	7	2	11	6	53	30
P5	Acute	1.5	0.08	0.06	1	1	0	1	0	43	26
P6	Acute	1	0.05	0.04	1	42	12 (group 1)	36	12 (group 1)	124	74
							5 (group 2)		2 (group 2)		
							1 (group 3)		1 (group 3)		

For P4, FLIPS analysis of two proviral genomes revealed that these sequences were genetically-intact and identical in the *gag*-3′ region but had changes outside of this region (Fig. 5B). One of these proviral sequences had a SNP outside of the binding site for the inner *gag*-3′ reverse primer but was still genetically-intact. The other proviral sequence had a deletion in stem-loop (SL) 2-3 of the *cis*-acting region, rendering the genome defective in the FLIPS 9.2-kb region but genetically-intact in the *gag*-3′ region used to sequence plasma-derived HIV-1 RNA (8.3 kb). Additionally, one proviral sequence from P6 with a premature stop codon was 99.98% identical to several genetically-intact proviral and plasma sequences in the *gag*-3′ region (Fig. 5D).

Although the number of identical sequences found within the paired plasma and PBMC samples was low, there did not appear to be any compartmentalization of the sequences derived from the PBMCs and plasma during untreated HIV-1 infection. For all participants, we observed that sequences from the PBMCs and plasma appeared to be interspersed on their phylogenetic trees (Fig. 4 and 5). This result was also supported by panmixia analysis showing a lack of genetic compartmentalization between the genetically-intact plasma-derived RNA and proviral DNA sequences within all participants (*P* > 10^−3^) (52, 53).

### Genetically mapping persistent HIV-1 within pre-ART and ATI samples.

**FIG 8:**
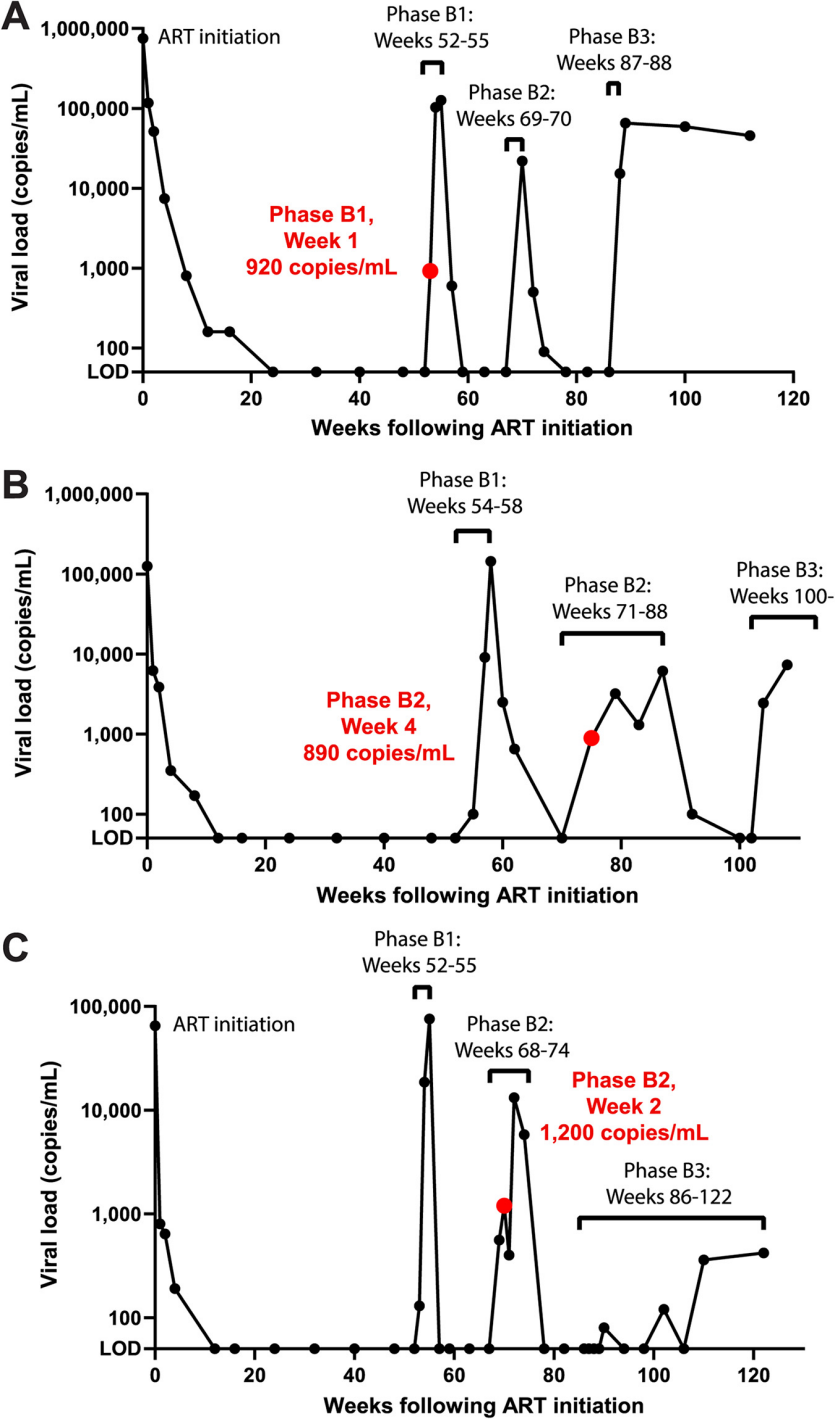
Viral load over the course of analytical treatment interruption study. Participants P3 (A), P4 (B), and P5 (C) initiated ART during acute HIV-1 infection, which they continued for 1 year. Each participant then interrupted ART for the first time (phase B1). After the indicated number of weeks, the participants reinitiated ART for 12 weeks and then interrupted ART a second time (phase B2). After the indicated number of weeks, the participants reinitiated ART and then interrupted ART for a third time (phase B3). Participants P3 and P5 reinitiated ART after the indicated number of weeks. The ATI plasma sample used for the PRLS assay *int*-3′ amplification is indicated by a red dot and text, with the viral load at this time noted. The limit of detection (LOD) is <50 copies/mL.

Previous studies sequencing short regions of the HIV-1 RNA genome have indicated that identical proviral sequences contribute to viral rebound during ART interruption (15, 17). To genetically-characterize a longer region of the rebound viral genomes, we used the *int*-3′ PRLS assay to amplify and sequence plasma-derived RNA genomes in ATI samples from three of the participants who initiated ART during acute infection (P3 to P5) (Table 2). These participants continued ART for 1 year before interrupting ART three times over the course of the following year (55). The collection time, viral loads, and number of amplified *int*-3′ sequences for the ATI plasma samples are shown in Table 2 and Fig. 8. We used the *int*-3′ PRLS assay to sequence plasma-derived genomes from these ATI plasma samples with lower viral loads to maximize the number of amplicons available for comparison to pre-ART sequences. To compare the *int*-3′ sequences amplified from the ATI plasma to the *gag*-3′ sequences amplified from pre-ART plasma and FLIPS sequences amplified from pre-ART PBMCs, we trimmed all sequences to the ∼4.7-kb region overlapping the *gag*-3′ and *int*-3′ amplicons (referred to as trimmed *int*-3′ region).

**FIG 9:**
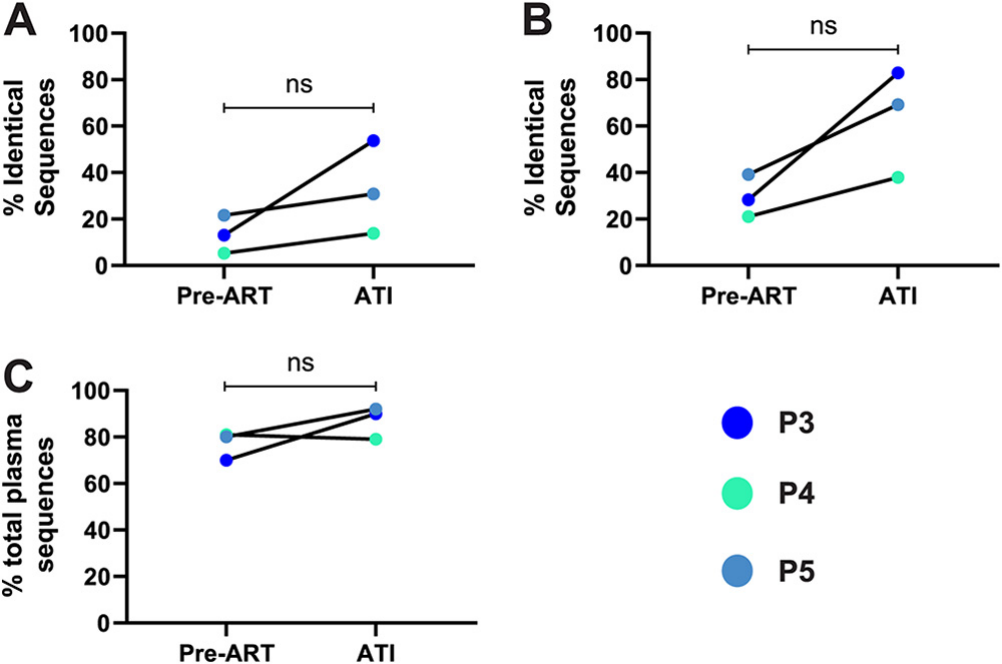
Properties of plasma-derived sequences from pre-ART and ATI plasma samples are similar. Plasma-derived sequences from pre-ART and ATI plasma samples for participants P3, P4, and P5 were trimmed to the ∼4.7-kb region spanning *int*-3′. (A and B) The proportions of sequences that were 100% (A) or >99.97% (B) identical to another sequence in the same sample were compared between the pre-ART and ATI plasma samples. A statistical comparison was performed using a Wilcoxon test and a paired *t* test, respectively. (C) The proportions of trimmed *int*-3′ plasma-derived sequences that are genetically-intact within the trimmed *int*-3′ region were compared between the pre-ART and ATI plasma samples. A statistical comparison was performed using a paired *t* test. ns, *P* > 0.05.

For all three participants, we observed that the proportion of 100% identical sequences within the trimmed *int*-3′ region was higher in the ATI plasma sequences than in the pre-ART plasma sequences, increasing from a median of 13% (IQR, 9.2 to 17.3%) in the pre-ART plasma to 30.8% (IQR, 22.3 to 42.2%) in the ATI plasma, though this did not reach statistical significance (*P* = 0.25) (Fig. 9A; Table 8). We also observed a trend that the proportion of trimmed *int*-3′ sequences that were >99.97% identical (allows 1 nt change between sequences) was higher in the ATI plasma sequences than in the pre-ART plasma sequences (pre-ART mean = 29.5%, ATI mean = 63.3%, *P* = 0.09) (Fig. 9B; Table 8). This shows that a large proportion of rebounding virions present during ART interruption are genetically-identical within the sequenced region, indicating that proliferating cells are contributing to the rebounding virus during an ATI.

**TABLE 8:** Identical pre-ART plasma-derived, pre-ART PBMC-derived, and ATI plasma-derived sequences within the same sample in the trimmed *int*-3′ region

Participant	Sample	% Genetic diversity of intact sequences in trimmed *int*-3′ region	% Proportion of sequences genetically-intact		Total no. of sequences	100% identical			>99.97% identical		
			Within sequenced region	Within trimmed *int*-3′ region		No. of sequences identical to another sequence	% of sequences identical to another sequence	No. of groups	No. of sequences identical to another sequence	% of sequences identical to another sequence	No. of groups
P3	Pre-ART PBMCs	0.07	76	81	37	9	24.3	2	23	62.2	1
	Pre-ART plasma	0.07	63	70	46	6	13.0	2	13	28.3	1
	ATI plasma (phase B1, wk 1)	0.09	90	90	41	22	53.7	5	34	82.9	5
P4	Pre-ART PBMCs	0.07	70	83	30	10	33.3	1	16	53.3	1
	Pre-ART plasma	0.11	63	81	57	3	5.3	1	12	21.1	1
	ATI plasma (phase B2, wk 4)	0.12	79	79	29	4	13.8	2	11	37.9	3
P5	Pre-ART PBMCs	0.06	85	92	26	3	11.5	1	15	57.7	1
	Pre-ART plasma	0.09	69	80	51	11	21.6	4	20	39.2	5
	ATI plasma (phase B2, wk 2)	0.10	92	92	39	12	30.8	6	27	69.2	7

Considering all three participants together, within the trimmed *int*-3′ region, the proportion of plasma-derived sequences that were genetically-intact did not differ significantly between the ATI plasma-derived sequences and the pre-ART plasma-derived sequences (*P* = 0.26) (Fig. 9C; Table 8). The mean proportion of genetically-intact plasma sequences in the pre-ART plasma for the three participants was 77% (95% CI, 70.1%, 83.9%), and the mean for the ATI plasma-derived sequences was 87% (95% CI, 79.1%, 94.9%). For all participants, the most common type of genetic defect was a frameshift mutation (Table 2). However, participants P3 and P5 did have a considerable increase in the proportion of genetically-intact plasma-derived sequences in the trimmed *int*-3′ region between the pre-ART plasma sequences and ATI plasma sequences, increasing from 70% to 90% for participant P3 and from 80% to 92% for participant P5 (Fig. 9C). The genetic diversity of genetically-intact sequences within the trimmed *int*-3′ region also did not change substantially between pre-ART and ATI plasma genomes (Table 8).

**FIG 10:**
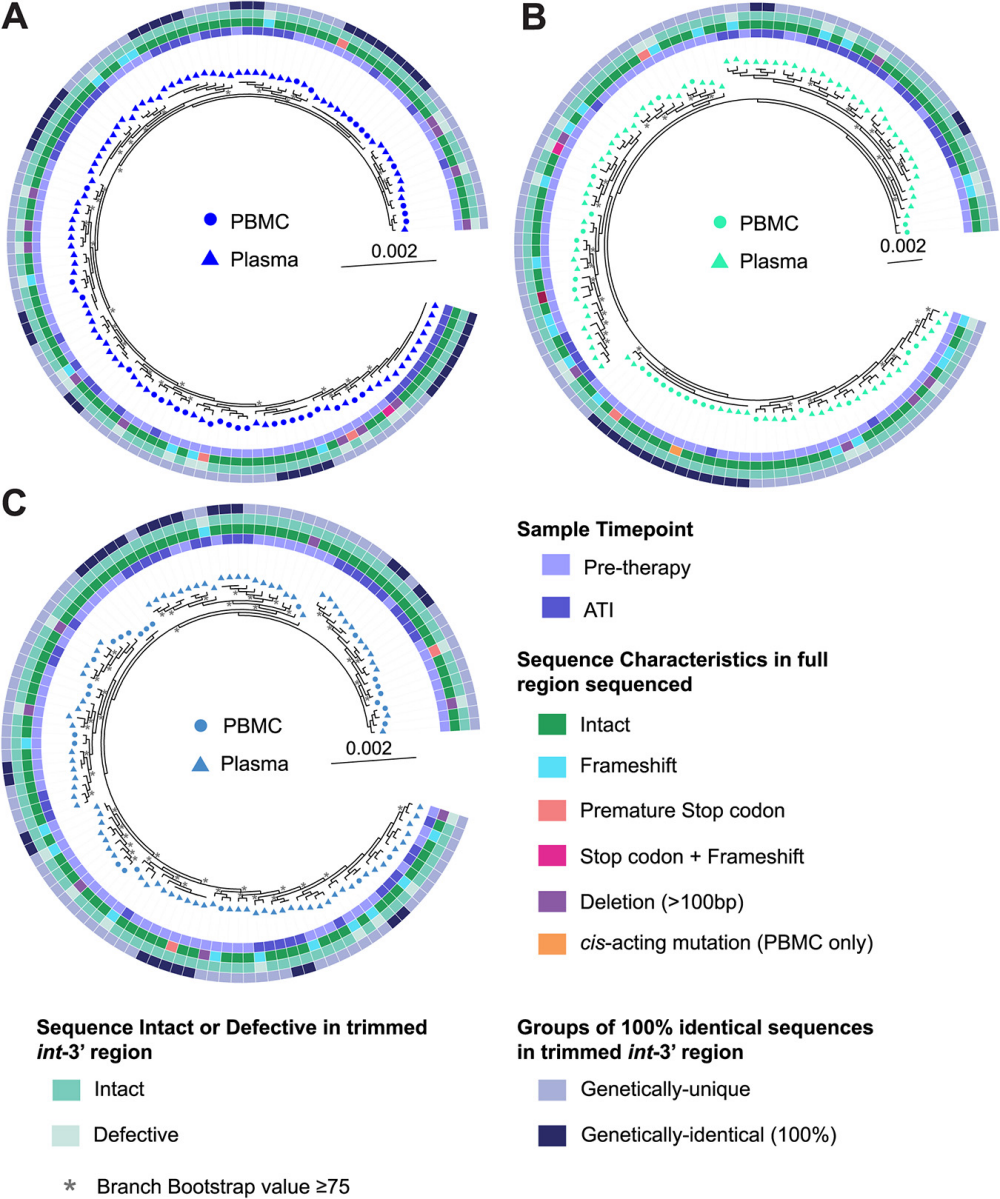
Phylogenetic trees of genomes from pre-ART plasma and PBMC samples and ATI plasma samples. Plasma-derived sequences from pre-ART and ATI plasma samples for participants P3, P4, and P5 were trimmed to the ∼4.7-kb region spanning *int*-3′ and aligned with trimmed proviral sequences from pre-ART PBMC samples, with sequences hypermutated or deleted in the trimmed *int*-3′ region excluded. Phylogenetic trees were then prepared for participants P3 (A), P4 (B), and P5 (C). PBMC-derived sequences are represented by circles, while plasma-derived sequences are represented by triangles. Inner rings show whether the sequence is from a pre-ART sample or an ATI sample. The second rings show whether a sequence is genetically-intact, or defective for each type of defect shown, within the full region sequenced for that sample (i.e., 8.3 kb *gag*-3′ for pre-ART plasma and 9.2 kb FLIPS for pre-ART PBMC). The third rings show whether a sequence is genetically-intact or defective within the trimmed *int*-3′ region. The outer rings show whether a sequence is 100% identical to another sequence in the tree in the trimmed *int*-3′ region. Branches with bootstrap values of ≥75 are indicated by an asterisk beside the node.

When we compared the pre-ART plasma and PBMC sequences to ATI plasma sequences for each participant, we found ATI plasma-derived sequences that were 100% identical to a pre-ART plasma sequence within the trimmed *int-*3′ region for all three participants (Fig. 10A to C). All sequences we identified as 100% identical to another sequence in the trimmed *int*-3′ region were genetically-intact in this region, though some were found to have genetic defects outside of this region (Fig. 10A to C). For participants P3 and P5, we found examples of clusters of multiple ATI plasma sequences that were 100% identical to a pre-ART plasma sequence (Fig. 10A and C). These pre-ART plasma sequences were genetically-intact in the full ∼8.3-kb *gag*-3′ sequenced region. Participants P3 and P4 also had an ATI plasma sequence that was 100% identical within the trimmed *int*-3′ region to a cluster containing both pre-ART plasma and PBMC sequences (Fig. 10A and B). For participant P3 (Fig. 10A), two of the pre-ART plasma sequences and two of the pre-ART PBMC sequences that contributed to this cluster were also found to be genetically-intact and 100% identical in their respective near-full-length sequences (i.e., 8.3-kb *gag*-3′ for plasma-derived RNA sequences and 9.2 kb for FLIPS PBMC proviral sequences). For participant P4 (Fig. 10B), two of the pre-ART plasma sequences were 100% identical to six of the pre-ART PBMC sequences in the *gag*-3′ region, four of which were also 100% identical and genetically-intact in the 9.2-kb FLIPS region. For this participant, two of the proviral sequences had defects outside the trimmed *int*-3′ region (Fig. 10B). Together, these findings indicate that many virions sequenced during an ATI are genetically-intact within the trimmed *int*-3′ region. Several ATI viral sequences were genetically-identical to viral sequences from pre-ART plasma and PBMC samples, indicating that HIV-1 reservoirs established prior to therapy can contribute to rebound virus.

## DISCUSSION

We have developed a novel method for near-full-length sequencing of plasma HIV-1 RNA by employing an SGS approach to amplify multiple individual ∼8.3 kb RNA genomes. We used the *gag*-3′ plasma-derived HIV-1 RNA using long-range sequencing (PRLS) assay to investigate the genetic composition of HIV-1 RNA within the plasma of eight ART-naive participants. In conducting this study, we found that 26 to 51% of viral particles present in the plasma of untreated HIV-1-infected individuals are genetically-defective.

Our studies of the HIV-1 RNA sequences from the culture supernatant of reactivated J-Lat cells revealed that the PRLS assay has a very low error rate of 0.0076%, or ∼0.63 nucleotides/*gag*-3′ genome. We found a higher rate of frameshift mutations when sequencing HIV-1 *gag*-3′ RNA genomes from the plasma of untreated participants compared to the *gag*-3′ sequences from the supernatant of J-Lat cells. In both the plasma of untreated HIV-1-infected participants and the J-Lat culture supernatant, the majority of these frameshift mutations were the result of 1- or 2-bp insertions or deletions (indels) in a homopolymer region of the HIV-1 genome. Indels within homopolymer regions can occur as a result of reverse transcriptase (RT)-PCR or sequencing error (56–59), though these indels can also be introduced during the HIV-1 replication cycle (47, 60–63). This indicates that the increased rate of frameshifts observed in the HIV-1 RNA from these participants is most likely the result of a combination of assay-related and viral replication errors. Since these mutations can be introduced during the HIV-1 replication cycle, we consider them genetic defects within the plasma-derived HIV-1 RNA.

This is the first study to quantify the proportion of genetically-intact and defective plasma-derived HIV-1 RNA genomes at a single-molecule level in untreated HIV-1 infection. We found that a median of 65% of plasma-derived HIV-1 genomes are genetically-intact during untreated infection, leaving 26 to 51% of plasma-derived genomes genetically-defective and therefore likely not infectious. The presence of defective proviral genomes in HIV-1-infected cells has been well-documented, both in untreated HIV-1 infection (64–67) and during effective ART (38, 64, 68), but it has generally been assumed that nearly all HIV-1 RNA genomes within plasma virions are genetically-intact. This is despite the fact that defective genomes have been observed when sequencing subgenomic regions of HIV-1 RNA from the plasma (19, 26–29, 69) and that *ex vivo* measurements of infectivity have revealed that most virions present *in vivo* are not infectious (70–72). The high proportion of defective genomes identified in our study using *gag*-3′ PRLS is consistent with the low level of infectious viral particles identified in these *ex vivo* measurements (1 tissue culture infective dose [TCID_50_] per 11,550 viral RNA copies) (71). Importantly, the defective genomes we have identified may contribute to the production of decoy HIV-1 proteins, which are known to be expressed from defective proviruses (73, 74). The host CD8^+^ T cell response to these decoy proteins contributes to HIV-1 immune evasion, chronic immune activation, and T cell exhaustion during ART (74, 75). These defective genomes may therefore have a pathogenic effect despite their presumed lack of infectivity. Recent studies sequencing subgenomic regions of plasma HIV-1 RNA have revealed that these sequences can match both genetically-intact and defective proviral sequences from HIV-1-infected cells (25, 64). This, in conjunction with our finding that defective genomes are present in the plasma of untreated HIV-1-infected individuals, indicates the importance of sequencing near-full-length HIV-1 RNA.

To ensure that our PRLS assay produced consistent results, we analyzed the plasma-derived *gag*-3′ HIV-1 RNA sequences in samples from two time points from the same participant during untreated chronic infection (P7), taken 27 days apart. The proportion of genetically-intact sequences and the proportion of each type of genetic defect were very similar between the two time points, and there was no evidence of sequence compartmentalization between the two time points. The viral genetic diversity has been shown to accumulate slowly during untreated chronic HIV-1 infection in more conserved genomic regions such as *pol* (43), while it may accumulate more quickly in more diverse regions such as *env* due to differing selection pressures (45). While we did observe a large difference in the genetic diversity of genetically-intact sequences within *gag* and *pol* compared to *env* for both time points, we did not observe a large change in the genetic diversity between TP1 and TP2 for any region. Overall, these results indicate the reproducibility of the PRLS assay and the slow accumulation of genetic diversity over 27 days of untreated chronic HIV-1 infection.

Genetic defects were found in all ORFs analyzed in this study, indicating the importance of considering the entire HIV-1 genome where possible when characterizing viral genomes in the plasma of HIV-1-infected individuals. We did, however, find an uneven distribution of defects across the genome, with a higher number of defects/genome found in *pol* and *env* than in *gag*. The 5′ half of the *gag* RNA genome has been shown to be important during packaging, allowing the selective incorporation of full-length RNA genomes into new virions during productive infection (76, 77). This may lead to a lower accumulation of mutations in *gag* during viral replication. We do acknowledge, however, that the *gag*-3′ PRLS assay does not sequence the first nine codons of *gag*, and mutations in this region are not identified. Nikolaitchik et al. demonstrated the importance of the first six nucleotides of *gag* in efficient packaging of RNA genomes by the HIV-1 Gag protein (78). Therefore, even though we did not sequence this region, it is reasonable to assume that this early portion of *gag* is intact, as the genomes were packaged into virions and released into the plasma. Alternatively, new virions require a functional Gag protein, and it has been demonstrated that Gag alone is able to assemble into a virus-like particle in culture (79). Therefore, the low proportion of plasma-derived genomes with defects in *gag* may reflect the fact that new virions are not able to be formed without a functional Gag protein. The increased proportion of defects found in *env* may reflect an increased accumulation of mutations in *env* in response to immune pressure, such as neutralizing antibodies (80–82) or CTL escape (83). This increased accumulation of mutations likely would also lead to an increased introduction of defects during viral replication. Further demonstrating this accumulation of mutations in *env*, we found a significantly higher genetic diversity within the *env* gene than in *gag* and *pol* within the genetically-intact plasma-derived *gag*-3′ sequences in our study of untreated HIV-1 infection. Furthermore, this immune pressure has also been previously shown to lead to mutations in *env* that can cause the virus to be less replication-competent, indicating that the presence of immune pressure can lead to genetic defects in HIV-1 *env* (84, 85). These findings highlight how sequencing subgenomic regions can greatly affect the observed genetic diversity and the presence of mutations within plasma-derived virions but not represent the true distribution of mutations across the genome.

We sequenced *gag*-3′ plasma-derived genomes with deletions of >100 bp, and 8% of these had break sites corresponding to the splice donor site D4 and splice acceptor site A7, which join *tat* and *rev* exons 1 and 2 in multispliced RNA transcripts (42). This suggests the presence of spliced RNA in plasma-derived virions, which has been demonstrated by *in vitro* transfection studies (86–88). However, this splicing of D4 to A7 does not occur without prior splicing of splice donor D1 to another downstream acceptor site (42, 89, 90). The deleted sequences we observed from the plasma reflect splicing of D4 to A7 alone, which could indicate that the presence of these genomes in the plasma are a result of another mechanism, such as extensive RNA secondary structure around the splice sites (91–94).

By employing the *gag*-3′ PRLS assay, we observed a relatively low proportion of identical plasma-derived RNA genomes during untreated HIV-1 infection compared to that of studies which sequenced subgenomic regions of the HIV-1 genome (8, 22, 46). However, we did observe identical plasma-derived RNA genomes for those participants who were at an earlier stage of infection or whose genetically-intact plasma-derived sequences had an overall lower genetic diversity. This indicates that a lower genetic diversity increases the likelihood of identifying identical sequences. We also found that identical genomes between the plasma and PBMC compartments during untreated infection were rare, though they were more common in participants that were in acute infection than in early infection. Despite the low number of identical sequences identified between the plasma and PBMCs, we found no evidence of compartmentalization between the two sites. The lack of identical sequences between these two sites could be attributed to the rapid turnover of HIV-1-infected cells during productive infection (95–97), an alternative anatomic source of some plasma virions (53, 96, 98), the depth of our sequencing limiting our ability to identify identical genomes, the sampled PBMCs representing only a low proportion of the circulating lymphocytes present at the time of blood draw (99), or the rapid introduction of mutations during active replication leading to multiple nucleotide differences between the RNA and DNA HIV-1 sequences.

For three participants whose peripheral blood samples were collected during untreated acute infection (P3, P4, and P6), we identified full-length (>8.25-kb) monotypic plasma-derived sequences that were 100% identical to at least two proviral sequences from their cells, and these identical sequences were genetically-intact within the sequenced *gag*-3′ region. For one participant (P6), we observed a large monophyletic group of 24 sequences which contained 12 monotypic RNA sequences and 12 identical proviral/DNA sequences. As the majority of cells in the peripheral blood contain one integrated HIV-1 DNA molecule, these findings suggest that multiple cells harboring genetically-intact and genetically-identical proviruses are contributing to plasma viremia (46). We cannot, however, exclude the possibility that these findings are the result of HIV-1-specific cells proliferating during ongoing viral replication (100). When we set the threshold for genetic identity at 99.98%, which allows a difference of 1 nt between full-length genomes, genetically-identical sequences between the plasma and PBMCs in the *gag*-3′ region were identified in all four participants whose peripheral blood samples were collected during acute infection. In identifying sequences that were >99.98% identical between the plasma and PBMCs in the *gag*-3′ region, we found only one case where a genetically-intact plasma sequence was 99.98% identical to a defective PBMC sequence (containing a premature stop codon in *pol*). No examples of defective plasma sequences being >99.98% identical to a full-length PBMC sequence in the *gag*-3′ region were identified. This indicates the importance of identifying genetically-intact HIV-1 RNA genomes for comparison with proviral genomes when investigating which HIV-1-infected cells contribute to viremia. To our knowledge, this is the first study to use near-full-length single HIV-1 plasma-derived RNA sequences to identify 100% or near-identical sequences between the HIV-1 plasma and proviral compartments.

For three participants who initiated ART during acute infection, we employed the *int*-3′ PRLS assay to compare plasma-derived RNA sequences from an ATI time point to pre-ART plasma-derived and proviral sequences trimmed to this region. Using the *int*-3′ PRLS assay for these ATI time points, we were able to maximize the number of sequences available for comparison with the pre-ART plasma-derived and proviral genomes. We observed that all plasma-derived RNA genomes from the ATI plasma that were 100% identical to another ATI plasma sequence or a pre-ART plasma or PBMC sequence were genetically-intact in the trimmed *int*-3′ region. However, it is important to note that the proportion of genetically-intact plasma-derived genomes in the pre-ART plasma increased from an average of 65% for these three participants to an average of 77% when the sequences were trimmed to the ∼4.7-kb *int*-3′ region, due to the mutations that rendered the genomes defective being found outside of the trimmed *int*-3′ region. Future studies will therefore require sequencing of ATI plasma samples using the *gag*-3′ amplicon to elucidate the contribution of virions containing genetically-defective genomes to viral rebound upon ART interruption.

Within the trimmed *int*-3′ region, we identified plasma-derived RNA sequences from ATI plasma samples that were 100% identical to pre-ART plasma-derived RNA sequences from all 3 participants. This is consistent with previous studies that have indicated that the viral quasispecies present pretherapy is similar to the quasispecies found during ART interruption (16, 54). Kearney et al. (15) also found examples of pre-ART plasma-derived sequences that were 100% identical to rebound plasma-derived sequences in a smaller region of the HIV-1 genome. These sequences were also found to be identical to variants that expressed cell-associated HIV-1 RNA during ART suppression, indicating their persistence and transcriptional activity during ART (15). Furthermore, we have identified plasma-derived RNA sequences in 2 of 3 participants from the ATI time point that were 100% identical within the trimmed *int*-3′ region to several pre-ART proviral and pre-ART plasma-derived sequences. Of note, a subset of these pre-ART sequences were 100% identical and genetically-intact in the longer regions sequenced using the *gag*-3′ PRLS assay and FLIPS. This suggests that cells integrated with this proviral sequence have persisted during ART and have contributed to viral rebound upon ART interruption, though we have not confirmed the presence of this sequence in proviral sequences obtained from samples during ART suppression. The contribution of proliferating cells to viral rebound during ART interruption and residual viremia during ART has been described previously (15, 17, 48, 101) but to our knowledge has not been demonstrated using an amplicon of this size. We note that there were several pre-ART plasma-derived and proviral sequences that were identified as 100% identical to this cluster of identical sequences within the trimmed *int*-3′ region but had mutations outside of the trimmed *int*-3′ region, some of which led to a premature stop codon or deletion in the *cis*-acting region of the genome. Future studies will require sequencing plasma-derived RNA from ATI samples using the *gag*-3′ amplicon to further elucidate the contribution of identical and intact proviral and plasma-derived sequences from pre-ART samples to rebound virions by determining whether the rebound viral sequences are truly clonal and/or match proviral sequences from proliferating cells.

Taken together, our findings highlight the *gag*-3′ or *int*-3′ PRLS assay as a more definitive method for identifying the cellular source of plasma viremia in studies of HIV-1 replication or rebound than methods that sequence shorter subgenomic regions of the RNA genome. Many ATI studies that sequenced shorter subgenomic regions in HIV-1-infected participants have been unable to identify a definitive cellular or anatomic source of rebound viremia due to low numbers of matching proviral and rebound sequences (25, 102–104) or that the rebound RNA sequences match proviral sequences from multiple anatomic sites within the small region sequenced (17, 105). The PRLS assay will allow a greater understanding of viral rebound during ATI by identifying the rebound RNA sequences that are genetically-intact, and therefore likely to be infectious, and by allowing the determination of the cellular source of these genomes. Sequencing the near-full-length genome from rebound virus provides additional insight into the contribution of recombinants to rebound viremia, as suggested by previous studies (25, 102, 104, 106). This indicates the great potential of the PRLS assay in identifying the cellular and anatomic source of genetically-intact plasma HIV-1 RNA genomes in viremic individuals.

However, we acknowledge several limitations of our study. First, the *gag*-3′ PRLS assay amplifies approximately 90% of the HIV-1 RNA genome, meaning that sequences that are identified as identical in this sequenced region may contain mutations in genomic regions outside of the sequenced area. However, this occurrence is predicted to be rare due to the large portion of the genome that is amplified using the *gag*-3′ PRLS assay. Similarly, sequences identified as identical by using the *int*-3′ PRLS assay may contain mutations in genomic regions outside of the sequenced area. Second, due to the extensive secondary structure in the 5′ untranslated region (UTR) of the HIV-1 RNA genome, we were unable to extend the region sequenced using the *gag*-3′ PRLS assay beyond the beginning of *gag*. This means that we are not able to sequence the *cis*-acting region of the HIV-1 genome, or the major splice donor (MSD, D1), and therefore determine whether defects in these regions, which are common in proviral sequences during effective ART (38, 64, 68, 107), are present in plasma-derived HIV-1 RNA genomes. Third, as we have not tested the *in vitro* replication competency of the sequences obtained in this study by viral outgrowth assay, we cannot be sure that the plasma RNA genomes identified as genetically-intact are truly replication-competent. Fourth, although we believe that RNA genomes with frameshift mutations due to small indels at homopolymer regions are present in the plasma, we are unable to distinguish the contribution of assay-related error and true HIV-1 replication-related mutation. Fifth, the *gag*-3′ PRLS assay can be used only on samples with high viral loads, as the instability of RNA molecules makes cDNA synthesis and PCR amplification of the ∼8.3 kb length inefficient. We have addressed this limitation by developing the *int*-3′ amplification, which is able to amplify and sequence plasma samples with lower viral loads, such as samples that are <350 copies/mL. Sixth, we have quantified only the proportion of defective HIV-1 plasma-derived RNA genomes in the near-full-length *gag*-3′ region in untreated participants with very high viral loads. It will be important to determine whether the high proportion of defective genomes remains in plasma samples with lower viral loads or whether the production of these virions with defective genomes is selected against during low-level replication. Finally, our observations regarding viral rebound during ATI were based on only three participants who initiated ART during acute infection, which may have increased our chance of identifying 100% identical sequences between pre-ART and ATI samples, due to the lower genetic diversity of genomes from participants who initiate ART during acute infection (50).

In conclusion, we have developed a new HIV-1 plasma RNA sequencing assay that enables the amplification and sequencing of near-full-length RNA genomes by using primers spanning *gag*-3′ (∼8.3 kb) or *int*-3′ (∼5 kb). We have used the *gag*-3′ PRLS assay to demonstrate that 26 to 51% of HIV-1 RNA genomes are genetically-defective in the plasma of HIV-1-infected individuals during untreated infection. Importantly, we identified near-identical sequences between the plasma and PBMC compartments in 4 out of 6 acute/early infection participants, and 3 of these participants had 100% identical sequences between these two compartments. By employing the *int*-3′ PRLS assay, we found that the majority of ATI plasma-derived sequences were identical to another sequence and genetically-intact. Several sequences from the ATI plasma samples were identical to viral sequences from pre-ART plasma and PBMC samples, indicating that HIV-1 reservoirs established prior to therapy contribute to viral rebound during an ATI. Our findings highlight the importance of sequencing long-read RNA transcripts at a single-molecule level to gain an accurate picture of the genetic landscape of plasma HIV-1 virions in studies of HIV-1 replication and persistence.

## MATERIALS AND METHODS

### Participant samples and study approval.

Stored plasma samples from 8 ART-naive HIV-1-infected participants were obtained for this study (Table 2). All participants were infected with HIV-1 subtype B. Six of these participants (P1 to P6), recruited through the Phaedra/Core01 AIEDRP protocol, had their blood draws taken during acute/early HIV-1 infection (1 to 4 months postinfection). For participants P1 to P6, cryopreserved PBMCs were also obtained from the same blood draw as the plasma for comparison (PBMC cell numbers ranging from 306,000 to 1,976,000 cells) (Table 2). Acute and early infection were defined as described previously (55).

For participants P3 to P5, stored plasma samples taken during an analytical treatment interruption (ATI) were also obtained for this study (Table 2; Fig. 8). Participants initiated ART during acute infection and continued treatment for 1 year. They then underwent three ATIs over the course of the following year, reinitiating ART for a period of time between, as described previously (55). Plasma samples with viral loads of ∼1,000 copies/mL were obtained from the first ATI for participant P3 and from the second ATI for participants P4 and P5 (Fig. 8).

Two participants (P7 and P8) had their blood draws taken during chronic HIV-1 infection (>12 months postinfection). P7 also had a second blood draw taken approximately 27 days after the first blood draw (time point 2 [TP2]).

Study approval was obtained from the institutional review board at the Western Sydney Local Health District, which includes the Westmead Institute for Medical Research. Phaedra/Core01 was approved through the St Vincent’s Hospital and University of New South Wales (UNSW) institutional review boards. All participants provided written informed consent.

### RNA extraction.

Up to 1.5 mL plasma was centrifuged at 5,300 × *g* for 10 min at 4°C to pellet any cellular debris present in the sample. The supernatant was transferred, and viral RNA extraction was performed as previously described, with minor modifications (18). Briefly, plasma was centrifuged at 21,000 × *g* for 1 h at 4°C. The supernatant was removed, and 60 µL Tris-HCl (pH 7.5) containing 200 µg proteinase K was added to the pellet. This was incubated at 55°C for 30 min. Two hundred microliters of 5.8 M guanidium isothiocyanate containing 200 µg glycogen was then added, and the mixture was incubated at room temperature for 5 min. Next, 270 µL 100% isopropanol was added, and the lysate was centrifuged at 21,000 × *g* for 15 min. The supernatant was removed, and the pellet was washed with 70% ethanol. The pellet was then dissolved in 35 µL 5 mM Tris-HCl (pH 7.5) containing 1 mM dithiothreitol (DTT).

For plasma samples diluted to viral loads of <100 copies, a second similar viral RNA extraction protocol was used as described previously (108), with minor modifications. Briefly, plasma supernatant was topped up to 9 mL with Tris-buffered saline, and samples were ultracentrifuged at 185,500 × *g* for 35 min at 4°C in a type 70.1 TI rotor (Beckman Coulter). The supernatant was then removed, and 100 µL 5 mM Tris-HCl (pH 7.5) containing 200 µg proteinase K was added to the pellet. Samples were incubated at 55°C for 30 min. Three hundred twenty-five microliters of 5.8 M guanidinium isothiocyanate containing 200 µg glycogen was then added, and samples were incubated at room temperature for 15 min. The lysate was transferred to a microcentrifuge tube, and 495 µL 100% isopropanol was added. The lysate was then centrifuged at 21,000 × *g* for 30 min. The supernatant was removed, and the pellet was washed in 70% ethanol and dissolved in 35 µL 5 mM Tris-HCl (pH 7.5) containing 1 mM DTT.

### cDNA synthesis.

Extracted RNA was split into two 16 µL reactions for cDNA synthesis. For each reaction mixture, the RNA was first treated with ezDNase (Invitrogen), according to the manufacturer’s instructions, with volumes of reagents doubled. This was to remove any contaminating DNA that was extracted alongside the RNA. Briefly, 2 µL 10× ezDNase buffer and 2 µL ezDNase were added to the 16 μL RNA, and the mixture was incubated at 37°C for 2 min. To inactivate the ezDNase, the mixture was then incubated at 55°C for 5 min in the presence of 10 mM DTT.

The cDNA synthesis was adapted from the protocol for the single-copy assay (108). Following DNase treatment, a 21.35 µL mixture consisting of 14 µM HIV-1-specific anchored oligo(dT) primer (7dT) (39) and 2.8 µM deoxynucleoside triphosphate (dNTP) (Invitrogen) was mixed with the RNA. The mixture was incubated for 65°C for 5 min, before it was immediately transferred to ice. A buffer mix consisting of 12 µL 25 mM MgCl_2_ and 6 µL 10× PCR buffer II (Invitrogen) plus 0.2% Tween was then added to the mix, followed by 40 U AffinityScript Multiple Temperature Reverse Transcriptase (Agilent Technologies). The cDNA synthesis cycling conditions were as follows: 42°C for 5 min, 55°C for 1.5 h, 70°C for 15 min, 4°C hold. The two separate reaction wells were pooled prior to amplification of the cDNA.

To quantify the viral load of plasma samples, cDNA synthesis was performed using random hexamers, as adapted from the protocol for the single-copy assay (108). Extracted RNA was treated as described in the section above, with the HIV-1-specific anchored oligo(dT) primer replaced with random hexamers (Promega) at the same concentration. The cycling conditions for the random hexamer cDNA synthesis were as follows: 15 min at 25°C, 42°C for 40 min, 85°C for 10 min, 25°C for 10 min, 4°C hold.

### PCR amplification of cDNA.

The nested PCR amplification as described previously (38) was adapted to amplify near-full-length genomes from cDNA synthesized from plasma of HIV-1-infected individuals. Briefly, cDNA was diluted to endpoint in molecular-biology-grade water before amplification using two rounds of a nested PCR as described previously (38). For the first PCR round, each 40 µL reaction mixture consisted of 2 µL diluted cDNA, 1 µM primers, 1× High Fidelity buffer (Invitrogen), 2 mM MgSO_4_ (Invitrogen), 0.2 mM dNTPs (Promega), and 0.025 U/µL Platinum Taq High Fidelity (Invitrogen). The first-round PCR products were then diluted 1:3 in Tris-HCl (5 mM, pH 8), and 2 µL was added to a 30 µL PCR mixture with reagent concentrations as described above. PCR cycling conditions were as described previously (38). The amplified cDNA positives were identified following visualization on a 1% agarose gel, with no exclusion of PCR products by size. The *gag*-3′ primers were RNA F1 (5′- TTTTGACTAGCGGAGGCT) and RNA R1 (5′- GCACTCAAGGCAAGCTTTATTGAGGCTTA) for the first round of the nested PCR, followed by RNA F2 (5′-GCGAGAGCGTCAGTATTAAGC) and RNA R2 (5′- CTGCCAATCAGGGAAGTAGCCTTGTGT) (67). The *int*-3′ primers were INT-3 F1 (5′- GGATTCCTGAGTGGGAGTTTG) and RNA R1 (5′- GCACTCAAGGCAAGCTTTATTGAGGCTTA) for the first round of the nested PCR, followed by INT-3 F2 (5′- CTTGGTAGCAGTTCATGTAGC) and GlobalR (5′- GCRGCTGCTTATATGCAGGATCT). The *int*-3′ forward primers (INT-3 F1 and INT-3 F2) were developed for the near-full-length amplification of HIV-1 proviral genomes using segments (64, 68).

### DNA extraction and amplification of proviral DNA.

Cryopreserved PBMCs from the same blood draws as the plasma samples were lysed using DirectPCR lysis reagent (Viagen Biotech) containing 0.4 mg/mL proteinase K, at an approximate concentration of 1 × 10^6^ cells/100 µL lysis buffer. The lysate was incubated at 55°C overnight (16 h) at 750 rpm using an Eppendorf ThermoMixer, followed by 95°C for 10 min at 750 rpm to inactivate the proteinase K.

Proviral DNA was amplified from lysed PBMCs by the full-length individual proviral sequencing (FLIPS) assay as previously described (38). The amplified products were identified following visualization on a 1% agarose gel, with PCR products of >8.9 kb length selected for sequencing.

### Sequencing and assembly of amplified genomes.

Amplified cDNA and PBMC positives were prepared for next-generation sequencing on the Illumina MiSeq platform as described previously (38).

HIV-1 genomes were assembled using either a workflow designed in CLC Genomics (Qiagen) as described previously for FLIPS (38) or a custom *de novo* assembly pipeline as described in a recent publication (109). Briefly, generated reads are first subject to QC trimming using BBDuk v37.98 (https://sourceforge.net/projects/bbmap/). Draft genomes are then assembled *de novo* using MEGAHIT v1.1.3 (110). Assembly of the genome is confirmed by mapping the reads onto the draft genome using BBMap v37.98. This is visualized using Geneious Prime v.2020.0.3, followed by extraction of the final majority consensus genome.

For genomes amplified from plasma-derived cDNA or lysed DNA from PBMCs, genetically-defective genomes were identified as those with inversions, deletions of >100 bp length, APOBEC3G-induced hypermutation, point mutations causing deleterious stop codons or missing start codons, or small (<100 bp) insertions or deletions (indels) causing frameshift mutations within HIV-1 ORFs (excluding *tat* exon 2 and *nef*) (38, 111–113). In addition, the *cis-*acting region, encompassing the packaging signal and major splice donor (MSD), was also assessed in the proviral genomes amplified from PBMCs. This was not assessed in plasma-derived genomes, as the PRLS assay does not amplify this region. Genomes with a deletion in the packaging signal or mutated MSD were classified as defective, though sequences with a mutated MSD could be salvaged by the presence of a cryptic splice donor found at HXB2 positions 748 to 749 (38, 114). Defects in ORFs were identified using the Los Alamos database tools GeneCutter (https://www.hiv.lanl.gov/content/sequence/GENE_CUTTER/cutter.html) and Hypermut (https://www.hiv.lanl.gov/content/sequence/HYPERMUT/hypermut.html), as well as the National Cancer Institute (NCI) Proviral Sequence Database (PSD) Proviral Sequence Annotation & Intactness Test (https://psd.cancer.gov/tools/tool_index.php) (115). For proviral sequences obtained from PBMCs, only sequences >8,800 bp in length, or those lacking deletions >100 bp in size, were included in the study.

### Further analysis of sequences.

Identical sequences were identified using the Los Alamos tool ElimDupes (https://www.hiv.lanl.gov/content/sequence/elimdupesv2/elimdupes.html) or by manually assessing matrices highlighting the number of differences between sequences, which were constructed from alignments using FastTree version 2.1.11 (116). A threshold of 100% identity was used to identify identical sequences, while near-identical sequences that differed by 1 nt were identified using a threshold of >99.98% identity for *gag*-3′ sequences and a threshold of >99.97% identity for sequences trimmed to the ∼4.7-kb region overlapping the *gag*-3′ and *int*-3′ amplicons. Sequences were classified as part of a cluster of >99.98% or >99.97% identical sequences if the sequence differed from another sequence in the cluster by 0 or 1 nt. To compare sequences amplified by PRLS and FLIPS, or to compare the number of clonal sequences identified by PRLS to that identified using SGS of short HIV-1 fragments such as *env* and *p6-RT* (8), alignments were trimmed to the primer binding sites yielding the shortest amplicon before additional analyses were performed.

Sequences were visualized using maximum likelihood phylogenetic trees constructed using FastTree version 2.1.11 (116), using the generalized time-reversible model, with gaps/deletions weighted by proportion of nongaps. Branch support values were calculated using 1,000 bootstrap replicates. Phylogenetic trees were visualized using ggTree (117), with the custom script described elsewhere (118). Hypermutated sequences were removed from alignments when phylogenetic trees were constructed. Genetic diversity of genetically-intact sequences was estimated by average pairwise distance (APD) using MEGA6 software (https://www.megasoftware.net/). APD was calculated when at least five genetically-intact sequences were available (28).

Tests for panmixia indicate the probability that two groups of sequences are two genetically-distinct populations (52, 53). We used a threshold of *P* < 10^−3^ to indicate evidence for compartmentalization between two different populations of sequences (52, 54). For the majority of participant samples, 20 randomly chosen genetically-intact sequences were compared per population, with sequences that were 100% identical excluded.

### Determination of error rate.

The reporter cell line J-Lat (clone 10.3) (40) was obtained from the NIH AIDS Reagent Program, Division of AIDS, NIAID, NIH. J-Lat cells are latently-infected with HIV-1, harboring one copy of HIV-1 proviral DNA per cell. Latency reversal was induced by incubating 5 × 10^4^ cells with 2 ng/mL phorbol 12-myristate 13-acetate (PMA) for 48 h. The culture supernatant from the activated cells was harvested and stored at −80°C until used for RNA extraction. Following RNA extraction and cDNA synthesis, 94 genomes were amplified and sequenced using the PRLS assay, and SNPs or frameshift mutations were identified in the genomes that were genetically different from the reference sequence for the J-Lat cell line (41). The number of nucleotides involved in these changes was quantified and divided by the total number of nucleotides sequenced (828,705 total nucleotides) to determine the mean and 95% CI for the error rate of the assay.

### Viral load quantification.

For participants P1 to P6, the viral load was determined by the Roche Amplicor kit 1.5 (Roche Diagnostics, Pleasanton, CA). Viral loads for participants P7 (TP1 and TP2) and P8 were quantified after cDNA synthesis, primed using random hexamers, using a droplet digital PCR (ddPCR) assay specific for the HIV-1 long terminal repeat (LTR) as described previously (119). The threshold was set using the ddpcRquant tool using the default parameters (120).

### Dilution of plasma samples.

To test the lower limits and reproducibility of the PRLS assay *gag*-3′ amplification, the viral load of participants P6 and P7 was used to dilute these samples so that they ranged from 1,000 to 10,000 copies per dilution, and each diluted sample was extracted and processed using the *gag*-3′ PRLS assay. Similarly, to test the lower limits and reproducibility of the *int*-3′ amplification, the viral loads of participants P6, P7, and P8 were used to dilute these samples to viral loads ranging from 10,000 copies to <50 copies per dilution. These diluted samples were then extracted using the appropriate extraction method and processed using the *int*-3′ PRLS assay. The plasma was diluted in 1× PBS (pH 7.4), RNase free (Invitrogen). For each diluted plasma sample, 10 µL of each participant plasma sample was extracted, and the number of viral copies was measured by ddPCR. This determined the actual number of copies of each diluted plasma sample that was sequenced. The lower limit of the assay was determined to be the lowest copy number for which an amplicon was obtained. A simple linear regression was calculated using Prism software (GraphPad) to demonstrate a positive correlation between the number of amplicons and the estimated copy number.

### Determination of *in vitro* recombination rate.

To determine the rate of assay-related intertemplate recombination introduced during reverse transcription, plasma samples from two different participants containing approximately 12,000 HIV-1 RNA copies were mixed prior to RNA extraction and *gag*-3′ sequencing. Plasma samples from each of the individual participants were also extracted and sequenced to obtain sequences for comparison to those from the mixed plasma sample. The number of extracted copies in each of these individual participant extractions was measured by ddPCR. From these individual participant extractions, it was determined that 13,304 copies from P6 and 11,001 copies from P7 were added to the mixed plasma extraction.

Two methods were used to investigate whether any intertemplate recombination was introduced during the mixed extraction and reverse transcription. All sequences obtained from the mixed extraction (87 sequences) were aligned to the sequences obtained from the individual extractions (P6, 48 sequences; P7, 56 sequences), and a phylogenetic tree was prepared from this alignment. Any *in vitro* recombination would be evident by sequences from the mixed extraction appearing separately to, but between, the sequences from the two extractions from the individual participant sequences. Clear separation was observed between the sequences from each participant, and the sequences from the mixed extraction grouped with only the individual participant sequences, indicating no evidence of intertemplate recombination.

We also used the RDP4 tool (121), using the default settings, to look for evidence of recombination between sequences from the mixed extraction and sequences from the individual extractions. A possible recombination event was identified if the event was predicted by at least 5 of 7 methods. Evidence of within-patient recombination was observed by multiple methods in participant P7, but no evidence of recombination between sequences from P6 and P7 in the sequences from the mixed extraction was observed.

### Statistics.

Statistical tests were performed using either Jamovi version 1.8.4 (122) (R Core Development Team [2020]), or STATA version 15 (123). A Kruskal-Wallis test was used to compare the proportions of defective plasma-derived sequences with each type of genetic defect, with pairwise comparisons performed using Dunn’s test with the Bonferroni multiple comparison correction. Mann-Whitney tests were used to compare the proportions of genetically-intact or defective plasma-derived sequences between the acute/early infection and chronic infection groups. A factorial analysis of variance (ANOVA) was used to compare the numbers of defects in plasma-derived sequences as a proportion of full-length genomes amplified in *gag*, *pol*, and *env* for the acute/early and chronic infection groups. The data were first log transformed (log_10_), and the normality of the transformed data was confirmed by constructing Q-Q plots (124). *Post hoc* tests for pairwise comparisons were performed using the Tukey protocol. The proportions of trimmed *int*-3′ sequences that were 100% or >99.97% identical to another sequence within this region were compared between the pre-ART plasma and post-ART plasma using a paired *t* test and a Wilcoxon test, respectively. The proportions of trimmed *int*-3′ sequences that were genetically-intact in this region were compared between pre-ART and ATI plasma samples using a paired *t* test. The normality of the data was determined using Shapiro-Wilk tests.

### Data availability.

All sequences analyzed in this study have been deposited in GenBank under accession numbers OK532372 to OK533278 and OM177064 to OM177172.
